# Flower-like meristem conditions and spatial constraints shape architecture of floral pseudanthia in Apioideae

**DOI:** 10.1186/s13227-022-00204-6

**Published:** 2022-12-19

**Authors:** Jakub Baczyński, Ferhat Celep, Krzysztof Spalik, Regine Claßen-Bockhoff

**Affiliations:** 1 Institute of Evolutionary Biology, Faculty of Biology, Biological and Chemical Research Centre, University of Warsaw, Warsaw, Poland; 2 Department of Biology, Faculty of Arts and Science, Kırıkkale University, Kırıkkale, Turkey; 3 Institute of Organismic and Molecular Evolution, Johannes Gutenberg-University, Mainz, Germany

**Keywords:** *Apiaceae*, *Cycloidea*, Floral unit, Flower, *Leafy*, Meristem, Morphogenesis, Pseudanthium, Ray flowers, *Unusual floral organs*

## Abstract

**Background:**

Pseudanthia are multiflowered units that resemble single flowers, frequently by association with pseudocorollas formed by enlarged peripheral florets (ray flowers). Such resemblance is not only superficial, because numerous pseudanthia originate from peculiar reproductive meristems with flower-like characteristics, i.e. floral unit meristems (FUMs). Complex FUM-derived pseudanthia with ray flowers are especially common in Apiaceae, but our knowledge about their patterning is limited. In this paper, we aimed to investigate both the genetic and morphological basis of their development.

**Results:**

We analysed umbel morphogenesis with SEM in six species representing four clades of Apiaceae subfamily Apioideae with independently acquired floral pseudanthia. Additionally, using in situ hybridization, we investigated expression patterns of *LEAFY* (*LFY*), *UNUSUAL FLORAL ORGANS* (*UFO*), and *CYCLOIDEA* (*CYC*) during umbel development in carrot (*Daucus carota* subsp. *carota*). Here, we show that initial differences in size and shape of umbel meristems influence the position of ray flower formation, whereas an interplay between peripheral promotion and spatial constraints in umbellet meristems take part in the establishment of specific patterns of zygomorphy in ray flowers of Apiaceae. This space-dependent patterning results from flower-like morphogenetic traits of the umbel which are also visible at the molecular level. Transcripts of *DcLFY* are uniformly distributed in the incipient umbel, umbellet and flower meristems, while *DcCYC* shows divergent expression in central and peripheral florets.

**Conclusions:**

Our results indicate that umbels develop from determinate reproductive meristems with flower-like characteristics, which supports their recognition as floral units. The great architectural diversity and complexity of pseudanthia in *Apiaceae* can be explained by the unique conditions of FUMs—an interplay between expression of regulatory genes, specific spatio-temporal ontogenetic constraints and morphogenetic gradients arising during expansion and repetitive fractionation. Alongside *Asteraceae*, umbellifers constitute an interesting model for investigation of patterning in complex pseudanthia.

**Supplementary Information:**

The online version contains supplementary material available at 10.1186/s13227-022-00204-6.

## Background

The remarkable architectural diversity of reproductive shoots works in tandem with floral morphology to maximize plant’s reproductive success [1]. As almost 90% of angiosperms [2] rely on biotic pollination vectors (insects and other animals), all flowers of an individual plant must act together to create an attractive display. The strong visual cue for pollinators can be achieved in various ways, one of them being formation of pseudanthia (aggregates of inconspicuous florets) that resemble and function like a single flower. Pseudanthia evolved independently among many lineages of angiosperms with diverse morphological patterns reflecting their varied phylogenetic descents [3]. Floral pseudanthia with the highest similarity to single flowers are characterized by flower-dimorphism. Peripheral flowers are enlarged and usually zygomorphic (‘ray flowers’), whereas the inner flowers are small and radial. Such a morphology is best-known from the heteromorphous heads of *Asteraceae* (such as daisies or sunflowers), but highly convergent units are also widespread among other campanulids [4, 5] which suggests the existence of specific developmental preadaptations for floral pseudanthia in this particular lineage of flowering plants.

### The diversity of pseudanthia in Apiaceae

**Fig. 1:**
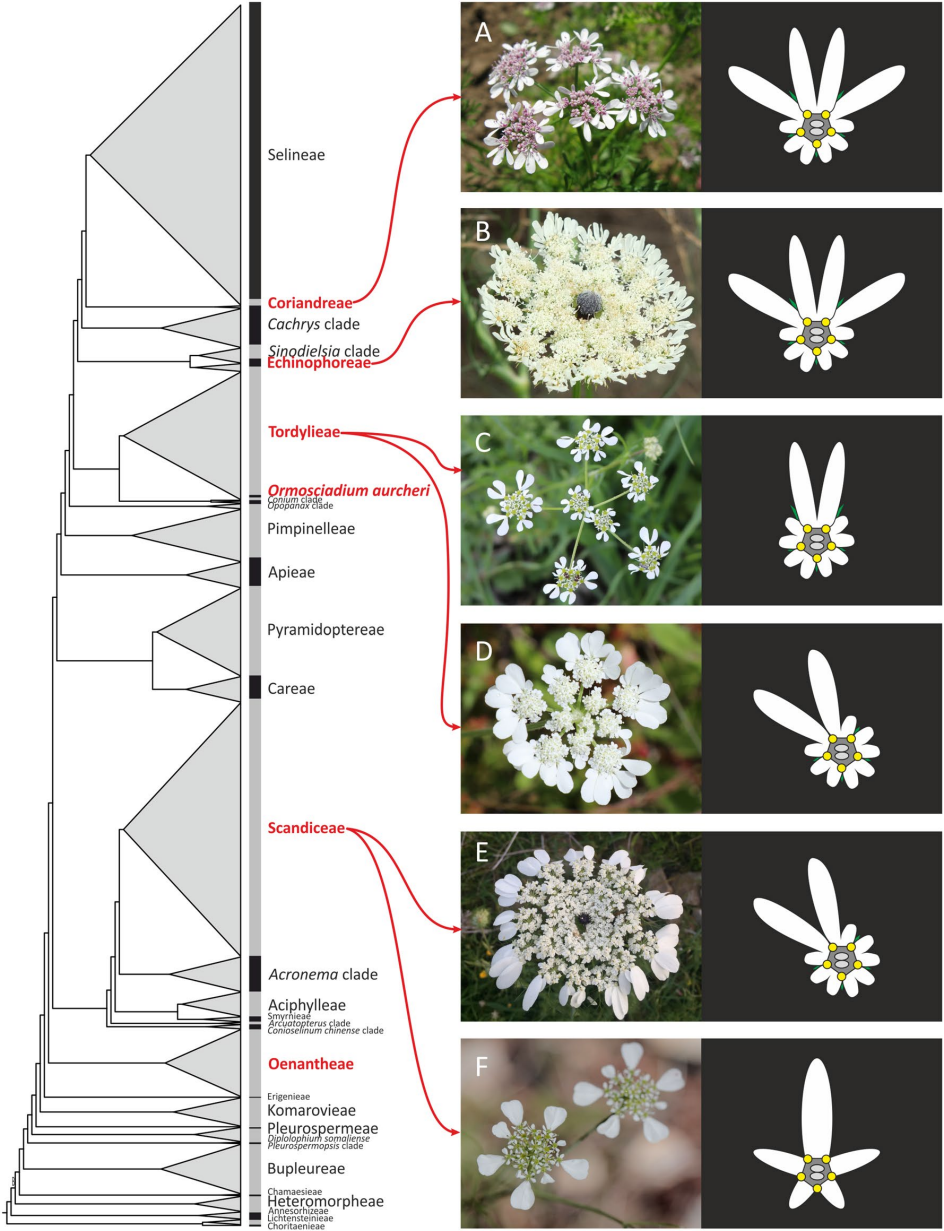
Simplified cladogram illustrating phylogenetic relationships of Apiaceae subfamily Apioideae and independent instances of origins of floral pseudanthia (red, modified after 58). Illustrations on the right visualize the diversity of species sampled for morphological investigation (with arrows indicating the clade in which the species is classified), including patterns of promotion and types of ray flower zygomorphy. **A** *Coriandrum sativum* with *Coriandrum-*type symmetry. **B** *Echinophora trichophylla* with *Coriandrum-*type symmetry. **C** *Tordylium apulum* with *Orlaya-*type symmetry. **D** *Tordylium apulum* with *Artedia*-type symmetry. **E** *Artedia squamata* showing *Artedia*-type symmetry of ray flowers. **F** *Scandix pecten-veneris* with *Scandix*-type symmetry

One of the notoriously understudied plant groups that repeatedly acquired floral pseudanthia is Apiaceae subfamily Apioideae (Fig. 1). This large, cosmopolitan clade of campanulid asterids, uniting over 3000 species is distributed across all continents except Antarctica. It comprises numerous economically important crops and aromatic herbs, such as carrot (*Daucus carota*), parsley (*Petroselinum* crispum), celery (*Apium graveolens*), cumin (*Cuminum cyminum*) and coriander (*Coriandrum sativum*). The diversity of pseudanthial forms in apioids results from fractal-like architecture of their unique complex umbels, i.e. umbels producing small umbels (umbellets). Pseudocorollas in Apioideae can develop around the entire umbel (umbel-centred promotion) forming a single pseudanthium (Fig. 1B, E), or around each umbellet (umbellet-centred promotion) resulting in several pseudanthia (Fig. 1A, C and F). Ray flowers of umbellifers are also diverse with respect to their pattern of zygomorphy that can encompass different numbers (and parts) of petals. These are often deeply winged due to apical inflexion of their primordia (forming a *lobulum inflexum* [6, 7]). The petal is symmetrical when the wings are equally developed or asymmetrical when the development of one wing is inhibited. Froebe [8] described three different types of zygomorphic pattern formations. The *Orlaya*-type has one enlarged, symmetrically winged petal (Fig. 1C). In the *Artedia*-type, two asymmetrical petals are enlarged and mirror images to each other (Fig. 1D, E). The *Coriandrum* type combines both forms by integrating three petals, a symmetrical one in the middle and two asymmetrical petals on its sides (Fig. 1A, B). Another feature of Apiaceae is the diverse initiation sequence of floral organ primordia during flower development. In different species, the sequence can be either centripetal, centrifugal with stamen dominance (they appear as first organs) or sectoral with groups of sepal, stamen and petal primordia appearing consecutively [9]. Despite all those information, until now, the developmental patterning of zygomorphy in ray flowers of Apioideae remains undescribed.

### Pseudanthia as floral units

The evolution of biological complexity is frequently based on build-up of simple structures into iterative, compound arrangements [10, 11]. This process is apparent in flower-bearing shoots which remarkable diversity is based upon a well-structured, modular architecture. In most angiosperms, an individual flower serves for the basic architectural module that can be born directly on a reproductive shoot (solitary flower) or as a part of a branched system, called inflorescence. An inflorescence meristem (IM) arises from a shoot apical meristem (SAM) and shares many of its qualities, such as apical growth resulting from the maintenance of stem-cell activity (caused by the maintenance of WUS–CLV3 regulatory loop) and ability to acropetally produce axillary meristems via segregation [12]. The self-perpetuation, growth and branching of IM ends with formation of ontogenetically determinate floral meristems (FMs). Depending on the timing and position of FM initiation, inflorescences can develop into simple determinate (botryoids) or indeterminate (racemes) units or their compound equivalents—botryoids of botryoids (compound botryoids/panicles) or botryoids and racemes of racemes (compound racemes).

Traditionally, all pseudanthia were termed inflorescences, as they are composed of numerous florets. However, recently, it has been recognized that alongside inflorescences (originating from IMs), individual flowers might develop as part of various lineage-specific, fundamental modules that repeat themselves on a reproductive shoot [11]. Some of these modules, collectively referred to as floral units, show flower-like developmental qualities. Their ontogenetically determinate meristems (floral unit meristems, FUMs) lack apical activity and instead expand, creating space for centripetal or centrifugal subdivision of submeristems [12, 13]. This process, known as fractionation, is controlled by local auxin maxima and proceeds until the entire surface of the FUM is used which implies that the initial size of the meristem and its intrinsic spatio-temporal constraints play substantial role in the patterning of floral units [14, 15].

FUMs are frequently associated with floral pseudanthia and constitute basic reproductive modules in several campanulid lineages, including umbels of Apiaceae [12], heads in Asteraceae [16] and Caprifoliaceae subfamily Dipsacoideae [17]. Unfortunately, our knowledge about the genetic patterning of floral pseudanthia is scarce and outside few model composites, the available data are restricted to genetic mechanisms underlying the elaboration of bilateral symmetry in ray flowers [18–20]. As proven by studies on *Gerbera* and *Helianthus*, development of the capitulum is governed by genes normally involved in the morphogenesis of single flowers [16]. For instance, its early developmental stages are characterized by the uniform expression of the conserved floral meristem (FM) regulator *LEAFY (LFY)* that marks it as a determinate structure [21]. Despite this profound change in the quality of the meristem, orthologues of asteracean *UNUSUAL FLORAL ORGANS* (*UFO*) genes retained their conventional function related to FM identity [21, 22]. The development of showy ray florets is controlled by orthologues of TCP genes *CYCLOIDEA* (*CYC*), normally implicated in the dorsal identity of monosymmetric flowers [23]. Asteraceae possess multiple paralogues of *CYC* that neofunctionalized creating different expression patterns in ray and disc flowers [24–27].

The numerous parallels between floral meristems and capitulum meristems indicate that acquisition of floral units may constitute an important prerequisite for evolution of pseudanthia in some plant lineages. The finding of similar molecular patterning in pseudanthia of plants that independently of Asteraceae acquired FUMs would provide further arguments for the formal recognition of floral units and the ‘ontogeny-based’ concept of inflorescences [13, 28]. Here, we present the results of our study focusing on developmental patterns in floral pseudanthia of Apiaceae. Our first aim was to describe how the processes of expansion and fractionation (idiosyncratic for FUMs and FMs) shape differences in the umbel- or umbellet-centred promotion of floral units and in the zygomorphy of their ray flowers. The analysis was based on six species, covering four phylogenetic lineages that independently evolved floral pseudanthia [29]. Our second aim was to analyse expression of three regulatory genes (orthologues of *UFO, LFY, CYC*) during the morphogenesis of umbels in wild carrot (*Daucus carota* subsp. *carota*) in order to answer the question whether floral units of Apiaceae show ‘flower-like’ regulation of development.

## Results

### Morphogenetic patterns in apioid pseudanthia

All analysed pseudanthia show developmental characteristics of floral units at both hierarchical levels (umbel and umbellet). Their patterning proceeds via expansion and fractionation, instead of apical growth and segregation that are to be expected from inflorescence meristems. Interestingly, Apiaceae with umbellet-centred ray-flower promotion and those with umbel-centred ray-flower promotion do not form two separate groups, but represents a continuum of pseudanthial morphologies with intermediate pattern visible in *Tordylium brachytaenium*. Ray flowers are initiated first, usually as common primordia with their subtending involucellar bracts. They are always perfect and developmentally accelerated in comparison with the inner radial flowers.

#### Umbel-centred ray-flower promotion

##### *Echinophora trichophylla* (*Coriandrum*-type zygomorphy)

**Fig. 2:**
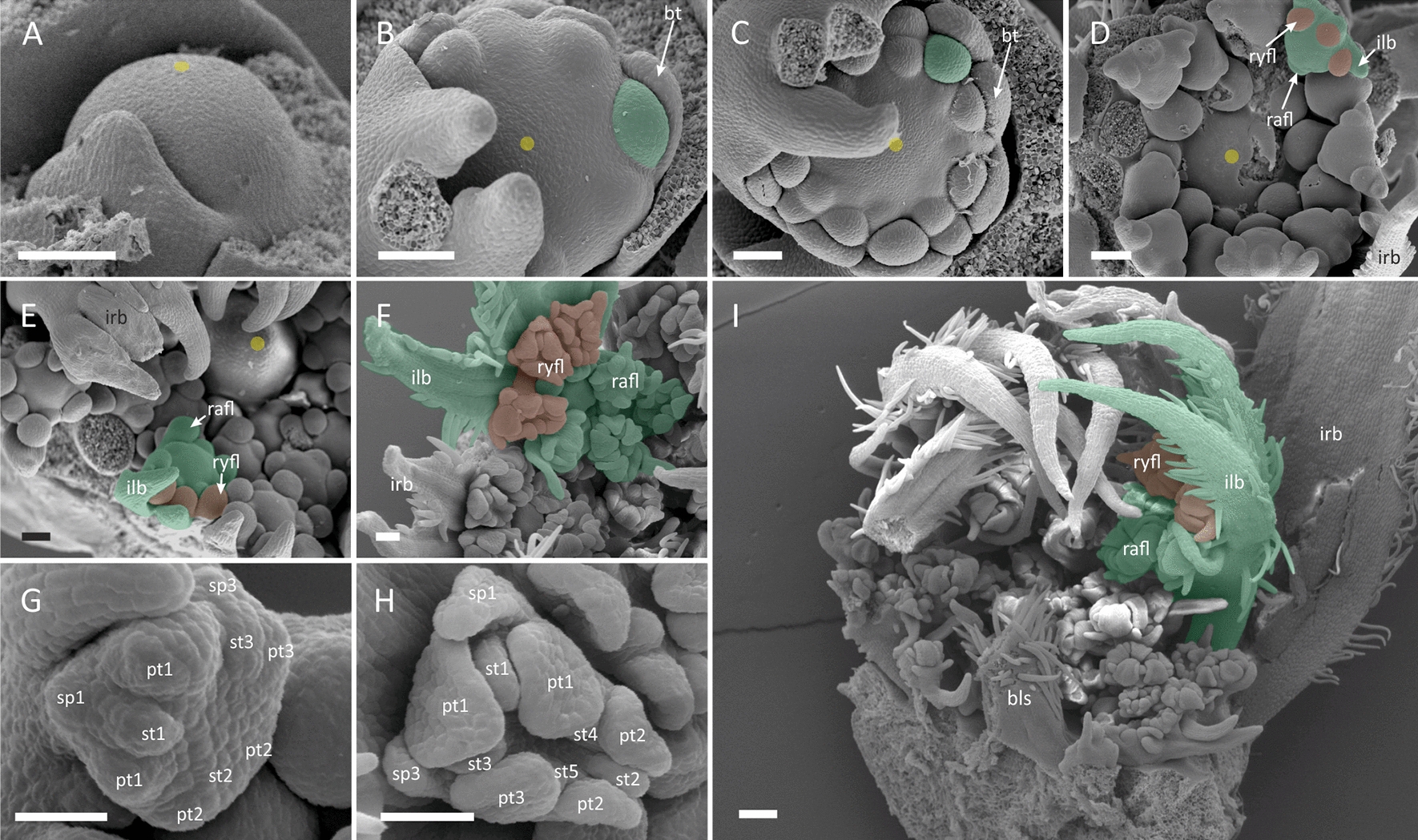
*Echinophora trichophylla.* Morphogenesis of the umbel (**A**–**F, I**) and ray flowers (**G, H**). To better visualize differences in promotion, a single umbellet from selected ontogenetic stages is marked with green shading with its ray flowers coloured red. **A** Large, naked FUM. **B** FUM fractionates peripheral umbellet meristems as common primordia with involucral bracts. **C** Central umbellet meristems follow. **D** Peripheral umbellets overtop central ones and fractionate common ray flower/involucel primordia. Note the persistent naked centre of the FUM. **E** Radial flower meristems follow. Note the bulging of the naked centre of the FUM. **F** Radial flower meristems fractionate floral organs. Ray flower meristems become zygomorphic **G** First floral organs are initiated from ray flower meristem in group-like patterns. **H** Ray flower meristem before gynoecium initiation. Note enlarged abaxial sepals (sp 1, 3) and zygomorphic symmetry of the meristem. **I** Late-stage of umbel development in longitudinal section. The naked centre of the FUM develops into a -plug-like structure. *irb* involucral bract, *umbt* umbellet, *ilb* involucellar bract, *rafl* radial flower, *ryfl* ray flower, *sp* sepal, *st* stamen, *pt* petal; *cp* carpel. Scale bars = 100 µm

Pseudanthia of *E. trich*ophylla develop on the level of the entire umbel (Figs. 1B and 2). The naked FUM (Fig. 2A) is large (ca. 500 μm) and centripetally fractionates numerous umbellet meristems that are initiated as common primordia with prominent involucral bracts (Fig. 2B, C). The centre of the FUM remains undifferentiated throughout the entire umbel morphogenesis (Fig. 2C, D and E), ultimately giving rise to a plug-like structure (Fig. 2I). Peripheral umbellets develop rapidly (Fig. 2D), overtopping the inner umbellets. The growth of the peripheral involucellar bracts is also accelerated. The pattern of floral meristem initiation in peripheral umbellets is unique. First, two ray flower/involucellar common primordia are fractionated at the abaxial side in some distance to each other. Then, a radial flower primordium appears at the opposite, adaxial side, leading to a distinctly triangular shape of the umbellet meristem (Fig. 2D). Next, the third ray flower meristem arises in the gap between the first two, also as a common primordium with an involucellar bract (Fig. 2D). Central umbellets show a different, spiral sequence of FM initiation (Fig. 2E). The incipient ray flower meristems are spherical and fractionate primordia in the group-like pattern. The first group arises from a common primordium at the abaxial side of the meristem and quickly divides into the primordia of dorsal and lateral petals, the dorsal sepal and its antesepalous stamen (Fig. 2G). Next, a similar group forms at the adaxial side of the meristem, fractionating the second lateral petal, second dorsal sepal and its antesepalous stamen. At the stage of gynoecium initiation (Fig. 2H) the meristem becomes zygomorphic and abaxial sepals elongate significantly into structures resembling ‘rabbit ears’ marking the onset of *Coriandrum*-type zygomorphy.

##### *Artedia squamata* (*Artedia*-type zygomorphy)

**Fig. 3:**
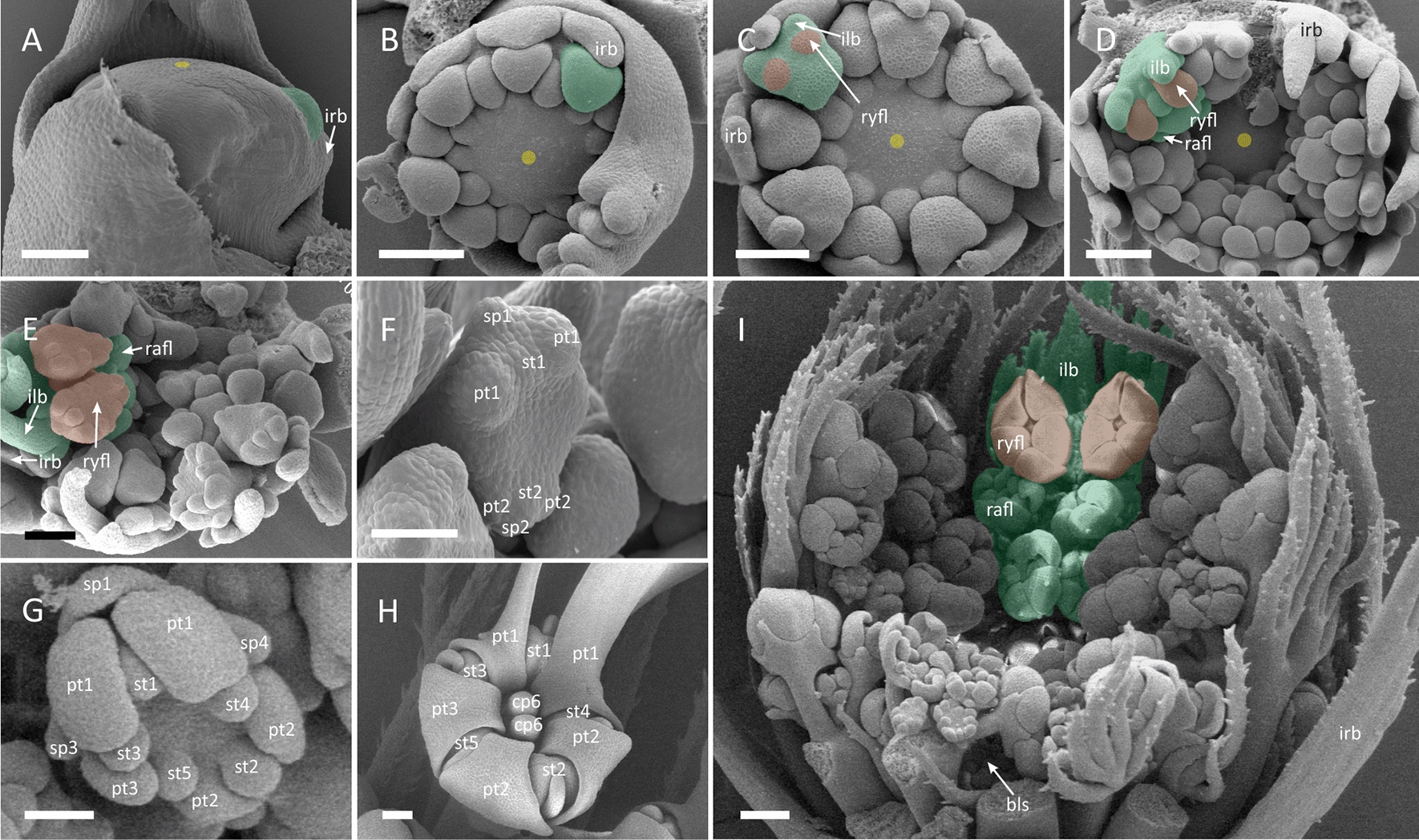
*Artedia squamata*. Morphogenesis of the umbel (**A**–**E, I**) and ray flowers (**F**–**H**). **A** Large and flat, naked FUM fractionates peripheral umbellet meristems as common primordia with involucral bracts. **B** Umbellet meristems follow in centripetal order. Note how involucral bracts enforce the change in peripheral umbellet meristems’ geometry**. C** Peripheral umbellets fractionate two common ray flower/involucel primordia. Central umbellets are developmentally retarded. **D** Umbel becomes cup-shaped. The naked centre of the FUM persists. **E** Pentagonal, mirror-imaged ray flower meristems develop in proximity to each other. **F** Ray flower meristem fractionating first floral organs in group-like pattern. **G** Ray flower meristem before gynoecium initiation. Note enlarged abaxial petals (pt 1) with interpetalous sepal and bifacial symmetry of the meristem. **H** Late-stage ray flower showing distinct, enlarged petal lobes (*Artedia*-type zygomorphy). **I** Late-stage of umbel development. For colours and abbreviations, see Fig. 2. Scale bars = 100 µm

Pseudanthia of *A. squamata* show an umbel-centred promotion of ray flowers (Figs. 1E and 3) and are virtually indistinguishable from these of *E. trichophylla* at the stage of umbellet fractionation (Fig. 3A). Its peripheral umbellets are also developmentally accelerated (Fig. 3I) at the umbel meristem which naked centre develops into brush-like structure. The largest difference between the two species is noticeable during the initiation of ray flowers. Initially, two ray flower meristems fractionate at the abaxial side of umbellets, followed by the radial flower meristem at the adaxial side. At the same time, involucral bracts are pressed against the gap between the first two ray flower meristems (Fig. 3B). The two ray flowers in each peripheral umbellet (Fig. 3C) develop rapidly getting oblique position, while their associated involucellar bracts push involucral elements away from the umbel (Fig. 3D). The initiation of floral organs follows the group-like pattern (Fig. 3F). The onset of the future *Artedia*-type zygomorphy (Fig. 3H) is established early, when ray flower meristems begin to press against each other, assuming mirror-imaged pentagonal shape (Fig. 3E, F).

#### Umbellet-centred ray-flower promotion

##### *Coriandrum sativum* (*Coriandrum*-type zygomorphy)

**Fig. 4:**
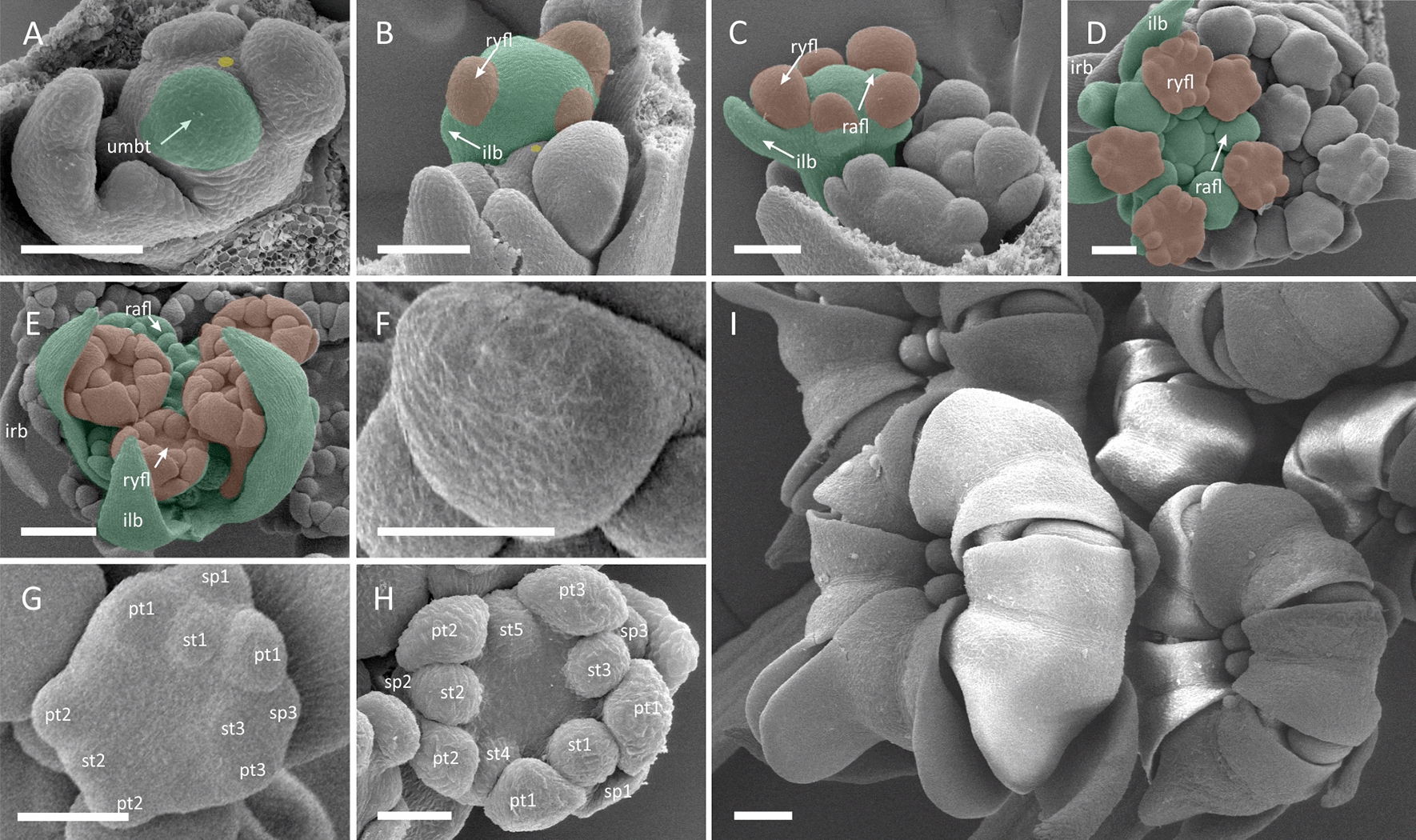
*Coriandrum sativum*. Morphogenesis of the umbel (**A**–**E**) and ray flowers (**F**–**I**). **A** FUM fractionates umbellet meristems. **B** The first initiated umbellet begins to fractionate common ray flower/involucel primordia in a spiral sequence. **C** Umbellets begin to elongate stalks (raylets) which results in their physical separation. When ray flower meristems are fractionating radial flower meristems follow in a centripetal sequence. **D** Ray flower meristems fractionate floral organs. **E** Ray flower meristems already develop the gynoecium when radial flowers start to fractionate floral organs. **F** Naked, spherical ray flower meristem. **G** First floral organs are initiated from ray flower meristems. **H** Ray flower meristem before gynoecium initiation. Note enlarged abaxial (sp3) and lateral (sp1) sepal. **I** Late-stage umbellet with ray flowers showing enlarged petal lobes. For colours and abbreviations, see Fig. 2. Scale bars = 100 µm

Pseudanthia of coriander show distinct, umbellet-centred pattern of promotion (Figs. 1C and 4). Their incipient FUM is small (ca. 100–150 μm) and becomes quickly occupied by few umbellet meristems which fractionate in a weak spiral pattern without associated involucral bracts (Fig. 4A). The development of umbellets proceeds centripetally with rapidly forming raylets (umbellets stalks) separating them vertically (oldest umbellet occupy highest position, Fig. 4B). At this stage, ray flowers are fractionated from umbellet meristems in a centripetal manner as common primordia with involucellar bracts (Fig. 4C), followed by bractless radial flower meristems (Fig. 4D). Involucels grow unevenly; those at the adaxial side of the umbellets are usually smaller (Fig. 4D, E) or even completely obsolete at maturity. The young ray flower meristems are spherical (Fig. 4F) in shape and fractionate floral organ groups (similar to those described in *E. trichophylla*) in a spiral sequence (Fig. 4G). After initiation of the first three floral whorls, the abaxial sepals enlarge quickly but remain visibly smaller than the petal primordia (Fig. 4H). Lastly, two carpels develop along the abaxial–adaxial axis of the flower indicating inferior gynoecium formation. At this stage, the *Coriandrum-*type zygomorphy of ray flowers is already established (Fig. 4I).

##### *Tordylium apulum* (*Orlaya*-type zygomorphy)

**Fig. 5:**
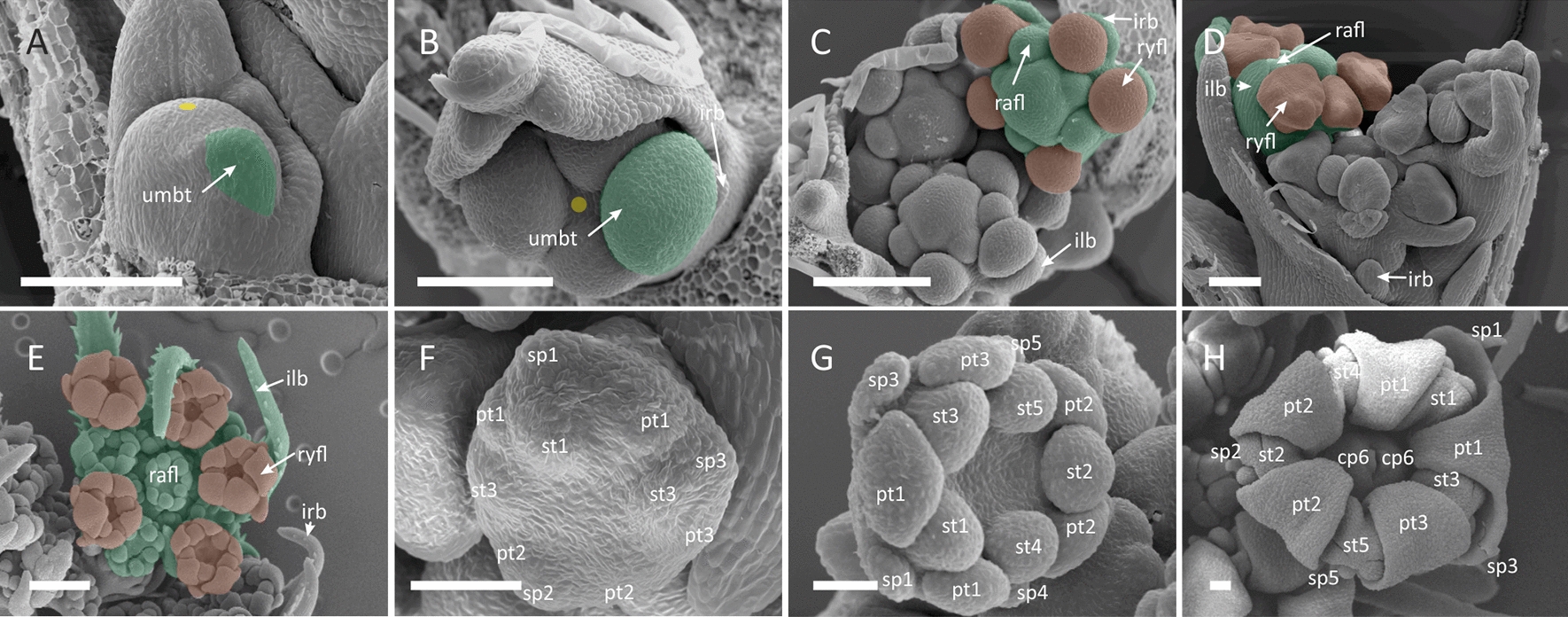
*Tordylium apulum*. Morphogenesis of the umbel (**A**–**E**) and ray flowers (**F**–**H**). **A** FUM fractionates the first umbellet meristem. **B** Subsequent umbellet meristems are initiated in a spiral pattern. Note the tiny primordium of the involucral bract. **C** Umbellets begin to elongate stalks (raylets) which results in their physical separation. **D** Ray flower meristems begin to fractionate floral organs. **E** Radial flower meristems fractionate floral organs when ray flowers start gynoecium formation **F** First floral organs are initiated from the naked, spherical ray flower meristem in a group-like patterns. **G** Ray flower meristem before gynoecium initiation. Note enlarged abaxial sepals (sp 1 and sp 3). **H** Ray flower bud showing enlarged abaxial petal lobes (*Orlaya*-type zygomorphy). For colours and abbreviations, see Fig. 2. Scale bars = 100 µm

The development of umbellet-centred pseudanthia in this species (Figs. 1C and 5) is almost identical to *Coriandrum* apart from the presence of small involucral bracts that subtend all or some of the umbellet meristems (Fig. 5B). The initial shape of ray flower meristems and their patterns of floral organ initiation are also reminiscent of the aforementioned species, despite differences in the resulting pattern of zygomorphy (Fig. 5H) which encompasses only abaxial petal and lateral sepals (*Orlaya*-type).

##### S*candix pecten-veneris* (*Scandix*-type zygomorphy)

**Fig. 6:**
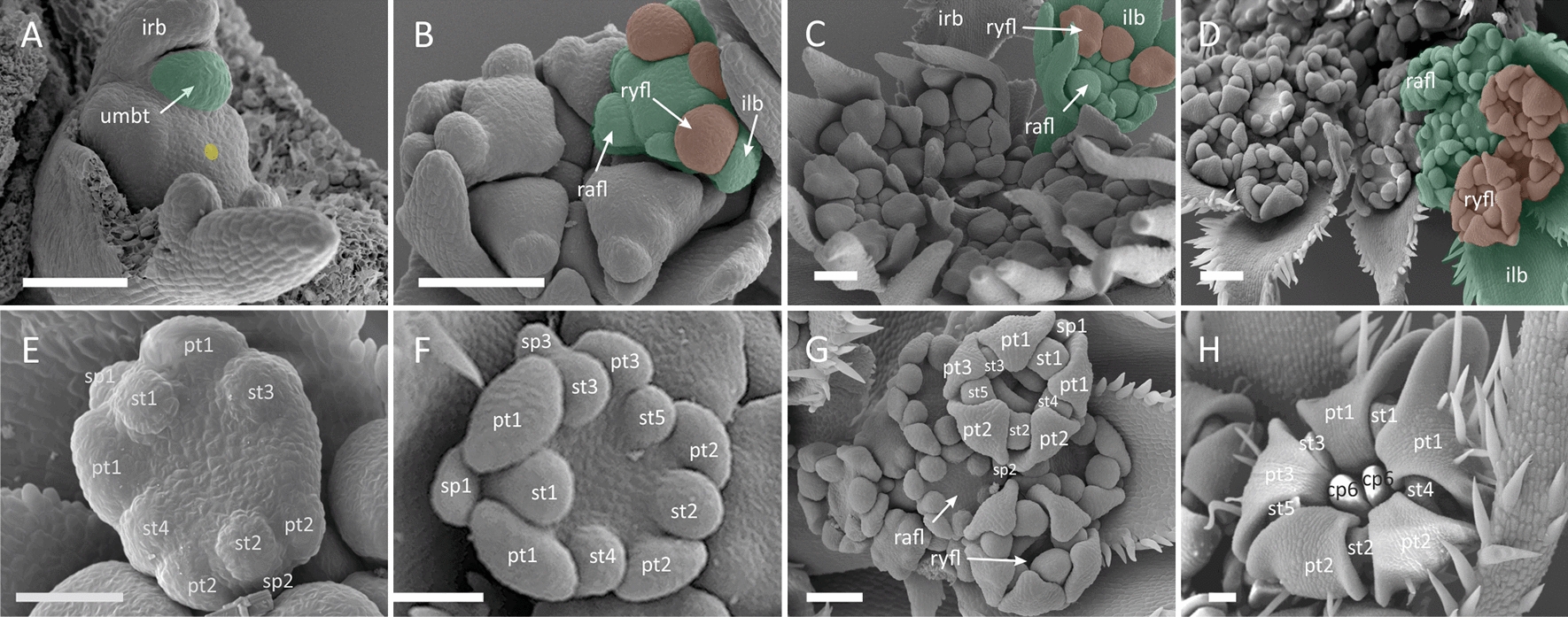
*Tordylium brachytaenium*. Morphogenesis of the umbel (**A**–**D**) and ray flowers (**E**–**H**). FUM fractionates umbellet meristems in a centripetal sequence. **B** Peripheral umbellets overtop central ones while fractionating FMs. Note the triangular shape of the umbellet meristems. **C** Ray flower meristems fractionate floral organs. Abaxial involucellar bracts are enlarged in comparison to adaxial ones. **D** Radial flower meristems fractionate floral organs. **E** First floral organs are initiated from the naked, pentagonal ray flower meristem. **F** Ray flower meristem before gynoecium initiation. **G** Peripheral umbellet with well-developed ray flowers not yet showing the enlarged petal lobes. **H** Ray flower bud showing enlarged abaxial petal lobes (*Artedia*-type zygomorphy). For colours and abbreviations, see Fig. 2. Scale bars = 100 µm

The ontogenetic patterns of pseudanthia in *S. pecten-veneris* (Figs. 1F and 6) deviate from all aforementioned species. Pseudanthia show extremely pronounced umbellet-centred promotion from the earliest stages of morphogenesis (Fig. 6B). While the first of the initiated umbellets fractionates ray flowers, younger ones are still at the naked phase (Fig. 6C). Elongation of raylets separates each of the umbellets into independent developmental units (Fig. 6E, I). Ray flower meristems develop as common primordia with associated involucels that enlarge rapidly into wide, bifid phyllomes (Fig. 6D). The sequence of floral organ initiation begins with stamens (Fig. 6F), followed by petals (Fig. 6G), and lastly—carpels (Fig. 6H). Sepals are obsolete throughout development. The zygomorphy of ray flowers is established very late and proceeds via elongation of the entire dorsal petal without forming enlarged petal wings (Fig. 6H).

#### Intermediate form of ray-flower promotion

##### Tordylium brachytaenium (*Artedia*-type zygomorphy)

**Fig. 7:**
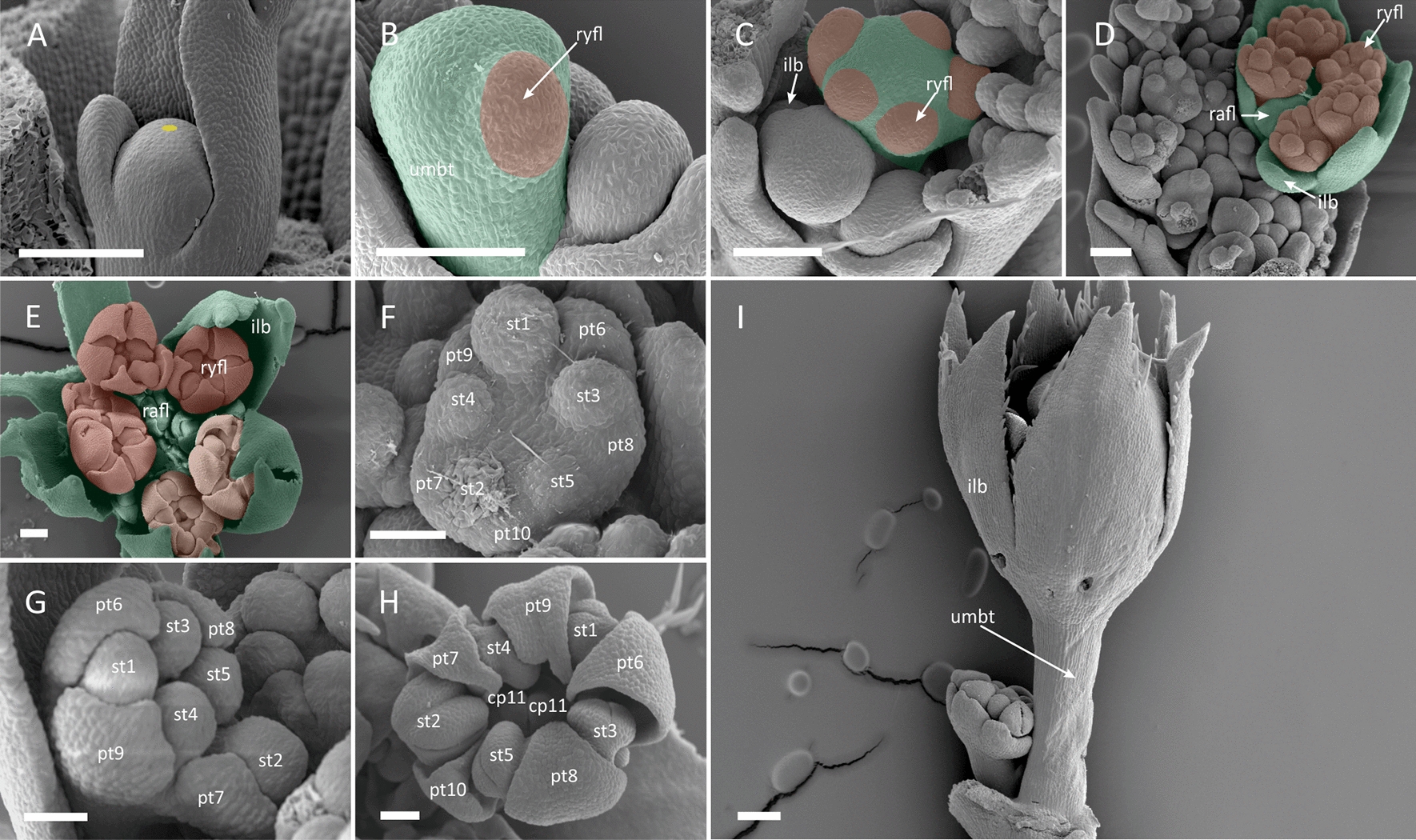
*Scandix pecten-veneris*. Morphogenesis of the umbel (**A**–**E, I**) and ray flowers (**F**–**H**). **A** Naked FUM. **B** FUM fractionates few umbellet meristems in a slow, successive manner. **C** Umbellets begin to elongate stalks (raylets) which results in their physical separation. The first initiated umbellet begins to fractionate common ray flower/involucel primordia in a spiral sequence. **D** The initiation of floral organs begins with stamen primordia. Prominent, bifid involucellar bracts become apparent. **E** Umbellet with mature ray flower buds and three radial flowers. **F** First floral organs (stamens) are initiated from spherical ray flower meristem in a spiral sequence. **G** Ray flower meristem before gynoecium initiation. Note the absence of sepals. **H** Late-stage ray flower during formation of gynoecium. The enlarged petal lobes have not yet developed. **I** Bud of a umbel. Note the enormous size discrepancy between the first and the third initiated umbellets (second one removed). For colours and abbreviations, see Fig. 2. Scale bars = 100 µm

An intermediate form of ray-flower promotion can be found in *T. brachytaenium* (Figs. 1D and 7). Its FUM is similar to that of the umbellet-promoted pseudanthia, but slightly larger (…µm). As the umbellet meristem size is almost equal, this additional space allows for the development of inner umbellets (Fig. 7A). The result is a superficially umbel-promoted pseudanthium with subperipheral umbellets showing smaller, frequently asymmetrical ray flowers (Fig. 7C, D). As in *E. trichophylla,* peripheral umbellets of *T. brachytaenium* pass through a triangular stage of development as result of the similar pattern of floral meristem initiation (Fig. 7B). At the early phases of morphogenesis, ray flower meristems stretch diagonally and become pentagonal, while floral organs fractionate in the already described group-like pattern (Fig. 7E). When carpels begin to differentiate, the outer sepal enlarges with two outer petals (Fig. 7F: sp1, pt1). Each of these petals forms only one wing next to the neighbour flower establishing *Artedia-*type pattern with two petals being mirror images to each other.

### Phylogeny of *CYC*-like genes

**Fig. 8:**
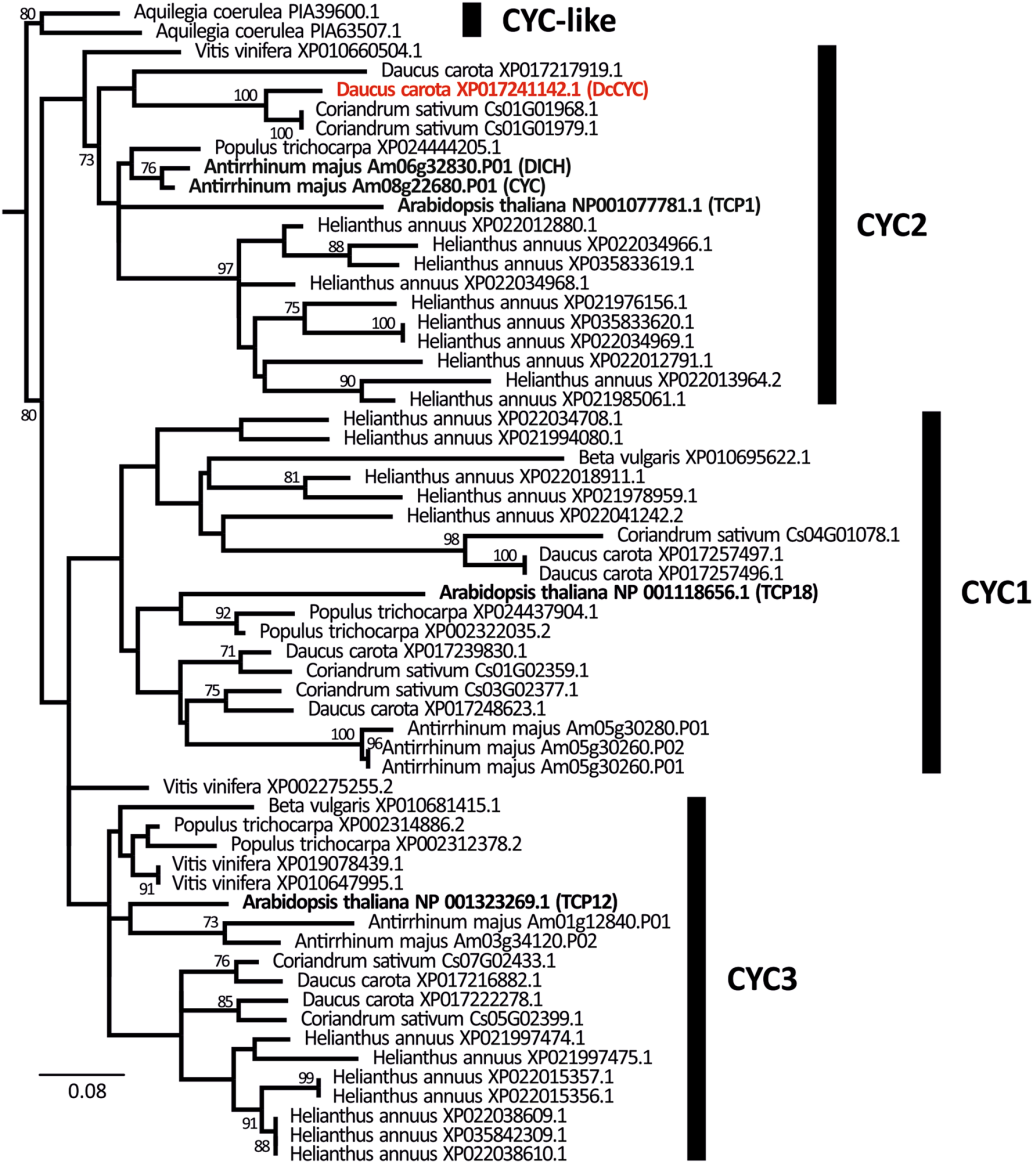
Maximum-likelihood tree of *CYC*-like genes based on amino acid sequences of conserved TCP and R domains. Major clades are defined with reference to canonical sequences (boldface). The sequence of *DcCYC* is marked with red. For visualization the tree was rooted with accessions of CYC-like genes from *Aquilegia coerulea* (early-diverging eudicot). Bootstrap values  < 70% were not plotted

The phylogenetic inference of *CYC*-like (Fig. 8) gene subfamily supports (with bootstrap = 80) its subdivision in *CYC*-like genes from early-diverging eudicots (represented by two accessions from the genome of *Aquilegia coerulea*) and a large, well-defined clade uniting CYC1, CYC2 and CYC3 gene types from core eudicots. In the latter group, only the monophyly of CYC2 genes is reinforced with bootstrap value of 73%. The lack of such support for the remaining clades may result from ambiguous position of *Vitis vinifera* sequence XP002275255.2 that is resolved in a polytomy with respect to CYC1 and CYC3 genes.

The CYC2 clade includes the canonical sequences of *CYC* and *DICHOTOMA* (*DICH*) genes from *Antirrhinum majus*, as well as TCP1 gene from *Arabidopsis thaliana*. The large expansion of this gene lineage is clearly visible in sunflower (the genome of which sports eleven paralogues of *CYC*). With respect to Apiaceae, both coriander and carrot possess two homologues of *CYC*, but based on available data it is impossible to unequivocally assess if they arose as a result of two independent or a single ancestral duplication. The carrot’s sequence XP017241142.1 constitutes a sister group (with bootstrap support of 100%) to two identical (with respect to TCP and R domain) paralogues from coriander. The second paralogue form carrot’s genome—XP017217919.1—is highly divergent from all umbellifer genes belonging to CYC2 lineage.

The phylogenetic reconstruction of *CYC*-like genes recovered multiple duplication events in asterid CYC1 lineage. The largest expansion occurred in sunflower (six paralogues), followed by carrot (four paralogues), coriander (three paralogues) and snapdragon (three paralogues). A single duplication is also recovered in black cottonwood, while the remaining rosids (*Beta vulgaris* and *Arabidopsis thaliana*) possess only one CYC1 gene. The already mentioned XP002275255.2 accession from *Vitis vinifera* may also belong to this clade. On the other hand, two paralogues from CYC3 clade were identified in most analysed species (cottonwood, wine, snapdragon, carrot and coriander). In this scenario, the single CYC3 genes in *Beta vulgaris* and *Arabidopsis thaliana* would arise because of secondary loss. Again, several events of duplication were recovered in sunflower with seven paralogues found in its genome.

### Gene expression patterns

**Fig. 9:**
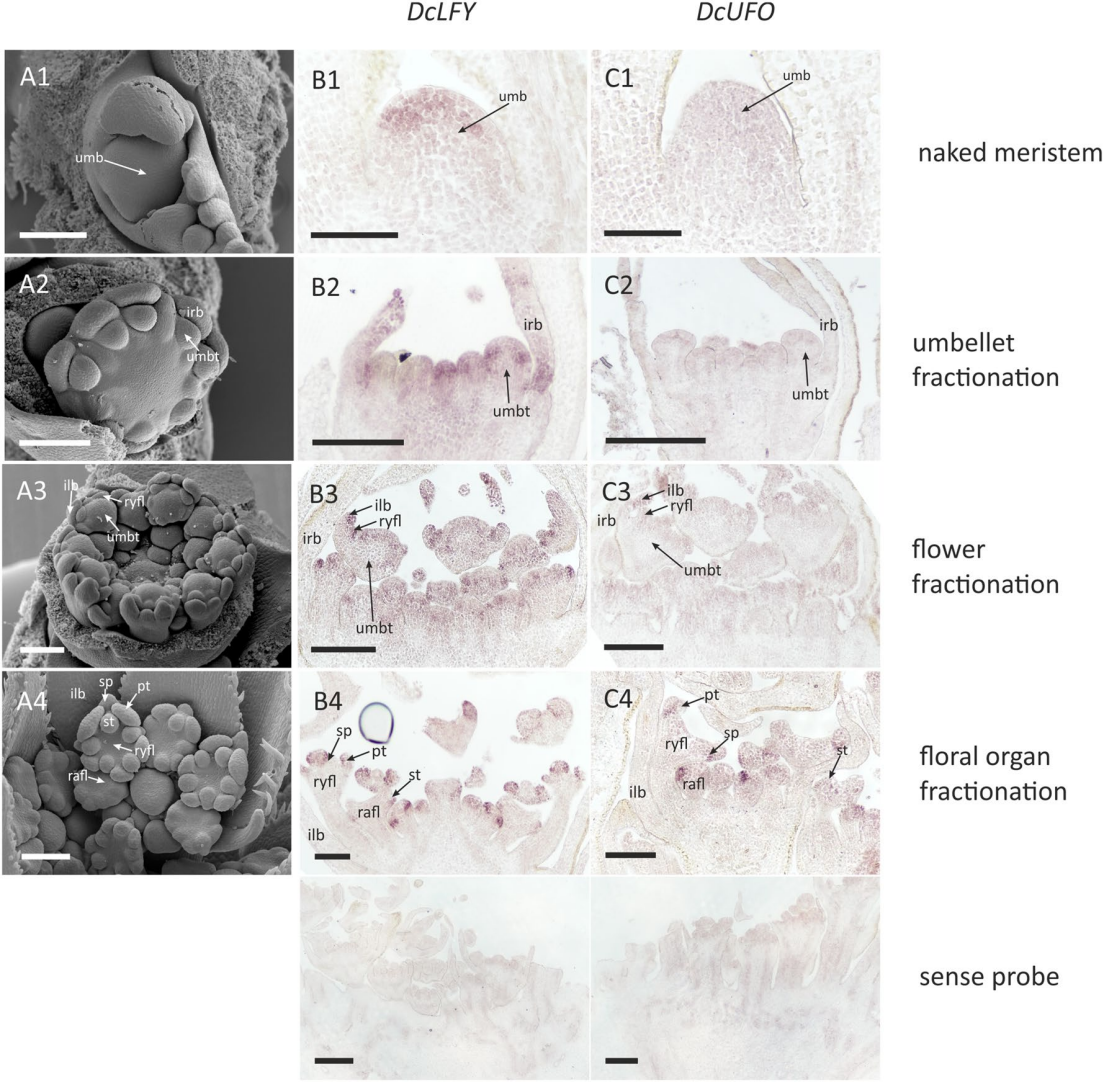
Expression of *DcLFY* and *DcUFO*. Letters correspond to: **A** umbel morphogenesis in *Daucus carota*; **B** *DcLFY*; **C** *DcUFO*. Note that the floral meristem identity gene *DcLFY* is repeatedly expressed on each meristem level: the umbel, umbellet and flower meristems. Numbers refer to developmental stages: **1** FUM; **2** initiation of umbellets; **3** fractionation of flower meristems from umbellet meristems; **4** initiation of floral organs (close-up on a single umbellet). Abbreviations see Fig. 2. Scale bars = 100 µm

The morphogenesis of umbel in carrot proceeds accordingly to other species with umbel-centred pattern of promotion (*A. squamata* and *E. trichophylla*). Its incipient FUM is relatively large and flat (Fig. 9A-1) and peripheral umbellet meristems are developmentally accelerated (Fig. 9A-2). Ray flower primordia are fractionated simultaneously with involucellar bracts at the adaxial side of umbellet meristems and enlarge faster than those of radially symmetrical florets (Fig. 9A-3). The pattern of future *Coriandrum*-type symmetry becomes apparent at the stage of carpel initiation (Fig. 9A-4).

*DcLFY* is expressed in the mantle zone of the entire FUM during earliest stages of umbel development (Fig. 9B-1). Afterwards, the gene can be detected in fractionating umbellets and flower meristems, as well as in the primordia of involucellar bracts (Fig. 9B-2). The expression of *DcLFY* is maintained in floral meristems and when floral organs begin to form (Fig. 9B-3) the gene’s transcripts can be localized in sepals and petals but not in stamens and carpels (Fig. 9B-4). Contrary to *DcLFY*, transcripts of *DcUFO* are absent from the first ontogenetic phases (Fig. 9B-1, 2, C-2) and can be found only in the common primordia of sepals, petals and stamens and later in sepals primordia (Fig. 9C-4).

**Fig. 10:**
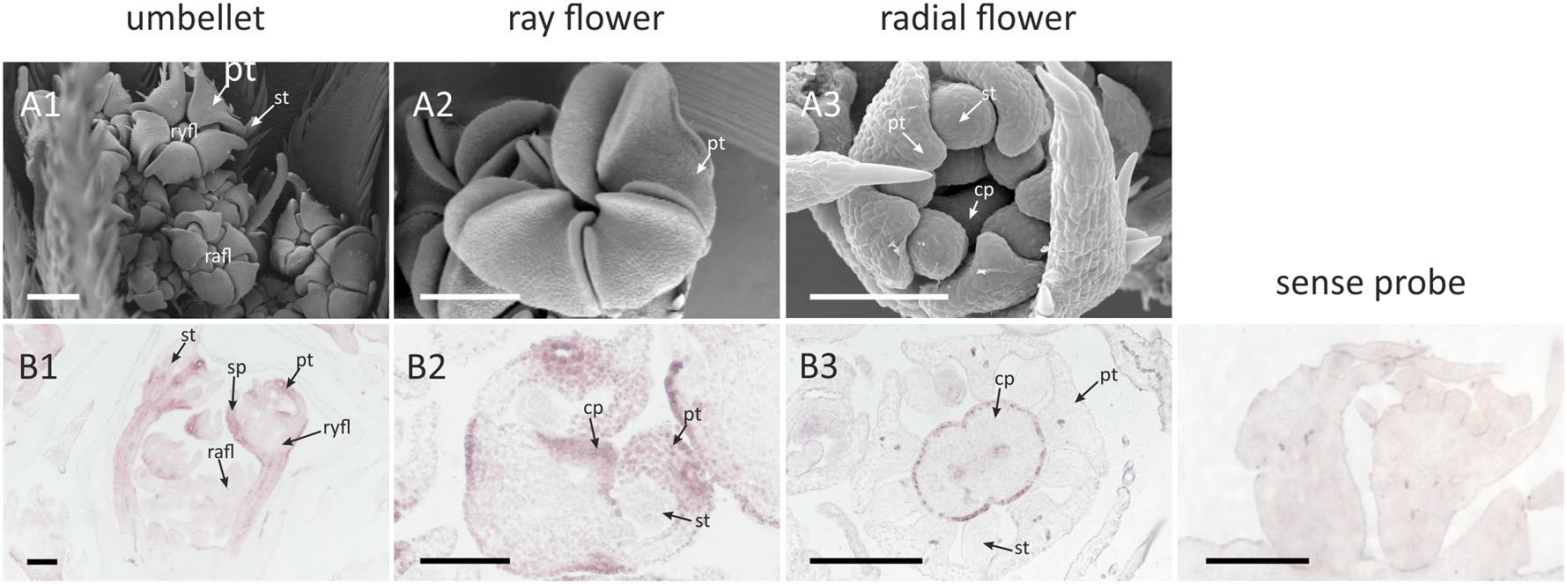
Expression of *DcCYC* in ray and radial flowers of *Daucus carota*. Letters correspond to: **A** flower morphology; **B** expression patterns of *DcCYC*. Numbers refer to: **1** entire umbellet; **2** ray flower; **3** radial flower. Abbreviations see Fig. 2. Scale bars = 100 µm

Based on the phylogenetic inference, we identified two paralogues nested within CYC2 clade (Fig. 8). In their case, the in situ hybridization experiments were focused on the latest developmental stage in which the asymmetry of ray flowers becomes apparent (Fig. 10A-1). After all organs are fully formed, *DcCYC* transcripts are found only in ray flowers (Fig. 10B-1), especially in the enlarged abaxial sepals and medial part of abaxial and lateral petals (Fig. 10B-2). In radial flowers, a weak expression can be traced to one or two peripheral layers of cells surrounding the gynoecium and medial part of the style (Fig. 10B-3). Although the second paralogous gene—*Daucus carota* XP017217919.1 (Fig. 8)—was expressed in dissected buds (which allowed for synthesis of probes) it could not be detected in developing umbels during in situ hybridization assays, despite several attempts with multiple variants of the basic protocol.

## Discussion

For a long time, internode inhibition in simple inflorescences was considered a universal path leading to the evolution of pseudanthia. For instance, capitula of Asteraceae were usually interpreted as derived from simple [30] or compound racemes [31], umbels [32], or spikes [33]. On the other hand, different forms of thyrses (inflorescences with primary racemose and secondary cymose branching) were suggested as the underlying architecture of the umbels in Apiaceae [34, 35]. Only recently, the detailed morphological reinvestigation of meristems and increased knowledge about genetic patterning led to the recognition of floral units [12, 13, 16]. Such multiflowered structures arise from determinate meristems resembling those of single flowers and, thus, are hardly comparable to conventional model plants which inflorescences can retain indeterminate growth for at least some time [36]. The ontogenetic prolongation of FMs, which we hypothesize as the possible pathway towards FUMs [37], may facilitate the co-option of various conserved developmental regulators (such as CYC/TB1 genes) from the level of individual flower to the level of entire floral unit and constitute an important preadaptation for the evolution of floral pseudanthia. As floral units are morphogenetically determinate, they cannot continuously segregate new submeristems/primordia due to apical growth. Their patterning is thus highly dependent on the space created by the initial and ongoing expansion of the FUM. Additionally, as apioid pseudanthia develop within the envelope of bracts and vegetative leaves that press them against the stem, their patterning is subjected to significant mechanical constraints.

### Floral pseudanthia in Apiaceae: the unique interplay among spatial constraints and morphogenetic gradients

Meristem geometry and mechanical constraints during morphogenesis can vastly alter plant morphology [38], including the number of organs [39], their proportion [40] and arrangement [41, 42]. Our understanding on how growth rate differences between cells influence gene regulatory networks during plant development is far from comprehensive and although gene expression can be induced solely by mechanical pressure (without secondary, auxin-mediated response), the mechanisms of such mechano-transduction remain elusive) [45, 46].

The importance of ontogenetic collisions for architecture of complex floral units has been explored with procedural modelling [47] and is vastly exemplified by experimental studies on simple heads and syncephalia of Asteraceae. Capitula primordia respond to wounding with a change of their phyllotactic pattern and only recently it has been proven that these alterations result from the disruption of natural auxin gradients [15]. In *Gerbera*, the release of spatial constraints from floral primordia adjacent to the wounding site allows for their repatterning into ray flowers. The bisection of a sunflower capitulum conducted by Marc & Palmer [45] almost four decades earlier yielded a similar outcome, i.e. the formation of two pseudanthial units with enlarged peripheral florets induced at the place of cut. Further proof for space-dependent patterning of FUM-derived pseudanthia comes from natural anomalies of syncephalus Asteraceae [46]. Contrary to the umbels in Asteraceae, syncephalia show distinctly enlarged ray flowers only at the periphery of the entire multi-headed unit. However, single capitula in *Oedera capensis* and *Dyssodia decipiens*, whose primordia were physically separated from their neighbours, are able to develop ray flowers around their entire margin [48].

**Fig. 11:**
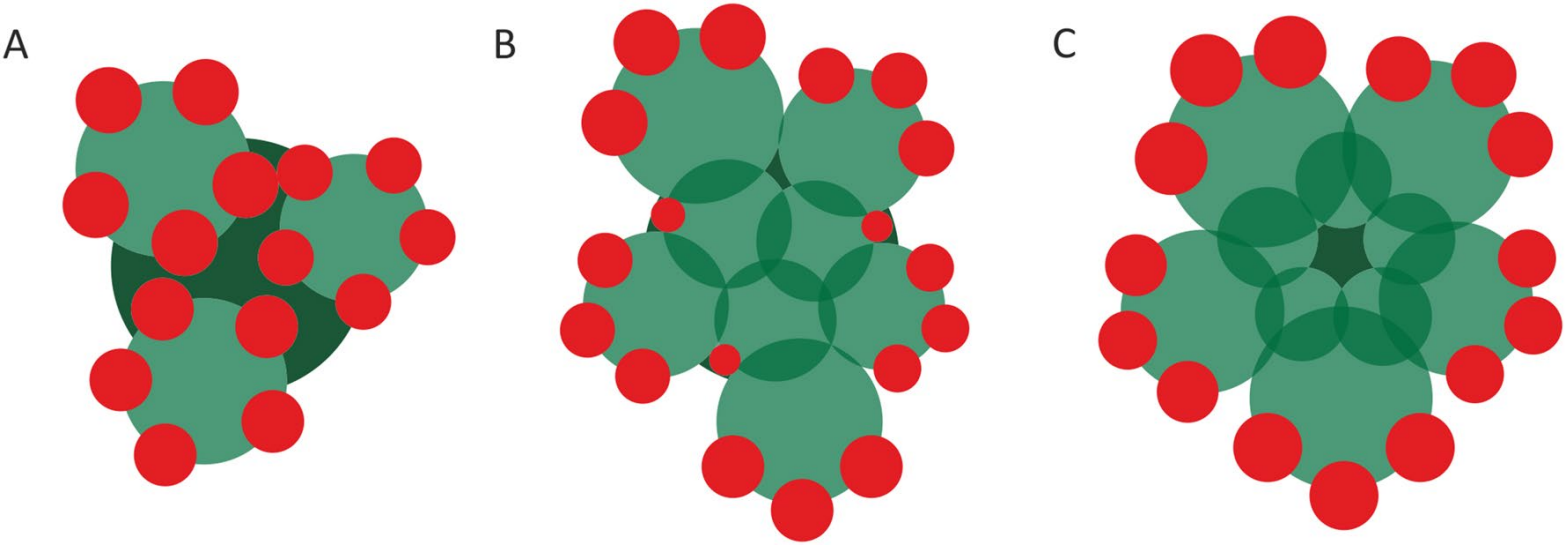
Theoretical model illustrating influence of spatial constraints on establishment of umbellet-centred (**A**), intermediate (**B**) and umbel-centred (**C**) promotion patterns in apioid pseudanthia. Green circles represent umbellet meristems. Red circles represent ray flower meristems. The overlapping areas between adjacent umbellet meristems indicate spatial constraints

Based on the aforementioned studies and results of our investigation, we hypothesize that patterns of ray flower formation in pseudanthia of Apiaceae result from the interaction between peripheral promotion and spatial constraints which increase towards the centre of the meristem (Fig. 11). Umbel shaping is based upon the relative size among the incipient FUM and umbellet meristems. Large FUMs give rise to umbel-centred units (Figs. 2 and 3). Spatial constraints are imposed by the numerous peripheral umbellets, which likely delay the fractionation of inner umbellet meristems and development of adaxial, ray flowers in the peripheral umbellet meristems (Figs. 2D, 3D and 11C). Thus, from the very beginning, the entire pseudanthium develops as a single entity, divided in a promoted peripheral and a retarded central part. On the contrary, when the FUM gives only rise to few umbellets, their physical separation caused by sequential elongation of stalks (raylets) releases mechanical pressure on the adaxial sides (Figs. 4, 5 and 6). In consequence, though all umbellets originate from the same FUM, each of them develops independently and forms ray flowers around its entire margin (Fig. 11A). The establishment of an intermediate promotion pattern in umbels proceeds almost identically to that of umbel-centred pseudanthia. However, due to the smaller size of the FUM and the corresponding smaller number of umbellets (Fig. 7A), its expansion goes along with a formation of additional space between the peripheral umbellets (Figs. 7C and 11B) allowing for the development of subperipheral units with smaller, weakly zygomorphic ray flowers (Fig. 7D). Interestingly, in Apiaceae, the promotion pattern might change within the individual plant (Additional file 1). The higher-order umbels (those that develop later on the reproductive shoot) are usually smaller and produce fewer umbellets. Species with umbel-centred promotion in strong terminal and first-order umbels can thus produce units with intermediate promotion in higher order as can be seen in carrot **(**Additional file 1**)**.

The influence of bracts on the polarity and growth of floral meristems is frequently overlooked in developmental studies [36, 49, 50]. It is widely acknowledged that size and position of bracts might be a source of spatial constraints that influence the shape of floral meristems and floral organ initiation patterns [51–56]. In Apioideae, the presence of bracts is highly variable but all species with floral pseudanthia have well-developed involucels [29]. They originate from FUMs as common primordia with ray florets and are thus not a part of the plant’s foliage (Figs. 2A and 3A). The peripheral promotion stimulus acts on those common primordia and causes their simultaneous outgrowth. In the result, similarly to ray flowers, apioid involucels are subjected to spatial constrains that depend on their position within the umbel and overall promotion pattern. In umbellet-centred pseudanthia, the influence of spatial constraints is noticeable in the developmentally retarded adaxial involucels which develop in direct contact with neighbouring umbellet meristems (Figs. 4, 5 and 6). In carrot, the high-order umbels with intermediately promoted ray flowers show a similar intermediate promotion in bracts (Additional file 1). The involucels of peripheral umbellets are large and distinctly pinnatisect, while those of central umbellets are noticeably smaller and needle-like. In subperipheral umbellets, enlarged bracts occur under weak ray florets and are also asymmetrical and less developed in comparison to those found in peripheral umbellets. Besides being subjects of spatial constraints, bracts in Apioideae can also create mechanical pressure themselves. In *Artedia squamata*, involucre-derived spatial constraints (Fig. 3B) on peripheral umbellet meristems cause retardation of one of the abaxial FMs, as well as changes in the geometry of its neighbours (Fig. 3C). The mechanical forces acting on the sides of ray flower meristems inhibit the enlargement in one of their lateral and dorsal petals, leading to the establishment of *Artedia-*type zygomorphy. In *Tordylium brachytaenium*, a similar effect is achieved by the proximity of adjacent FMs (Fig. 7C and E) which press against each other. The pressure-dependent shift in ray flower meristem symmetry (from radial to zygomorphic) is also apparent in *Echinophora trichophylla* (Fig. 2H), however its occurrence at late developmental stages—after petals, sepals and stamens are well-developed—does not cause a distortion in the patterning of *Coriandrum*-type zygomorphy. This observation implies that ontogenetic spatial constraints may have different effects on ray flower morphogenesis, depending on the timing of their occurrence (early or late shift in symmetry sensu Naghiloo, [57].

### Floral unit meristems—an important preadaptation for pseudanthia?

The negative feedback loop of CLAVATA3 (CLV3) and WUSCHEL (WUS) constitutes a key genetic component of stem-cell activity, accounting for self-perpetuation of IM and formation of cellular pool necessary for proper development of flowers [58]. While WUS promotes cytokinin activity in the central zone of IM and incipient FMs, CLV3 restricts meristems’ size by preventing the build-up of excess cells [59]. The disruption of WUS–CLV3 loop is necessary for cell differentiation and organogenesis. In *Arabidopsis thaliana*, when a certain size of the floral meristem is reached, WUS acts with LEAFY (LFY) to activate its own repressor—AGAMOUS (AG) [60]. This process establishes the determinacy of the future flower. Other transcription factors that confer floral fate include *UFO* which works in combination with *LFY* to specify petals and stamens by activation of B-class MADS box genes [61, 62].

FUMs differ from inflorescence meristems and instead resemble flower meristems. Similar to FMs, FUMs are characterized by the early determinacy and lack of apical growth which in Asteraceae coincides with uniform expression of *LFY* [21] homologue in the naked (i.e. undifferentiated) head meristem. According to our results, umbels in Apiaceae share this pattern (Fig. 9B-1). In carrot, the transcripts of *DcLFY* can be detected at the umbel meristem and throughout the process of repeated fractionation in the umbellet meristems and flower primordia (Fig. 9B-2, 3). This indicates that floral units, such as flowerheads and umbels, ontogenetically resemble ‘matryoshka dolls’, i.e. or ‘umbels within umbels and that the simple process of internode inhibition cannot sufficiently explain their unique developmental patterning. Following Claßen-Bockhoff and Frankenhäuser [37], we hypothesize that FUMs might arise from FMs due to disruption of WUS–CLV3 signalling pathway, including the loss of size-restricting function of CLV3 and/or lack of negative feedback from other direct/indirect repressors of WUS. Such a change would result in the expansion of the meristem and creation of space for additional fractionation, however, this hypothesis requires further studies to be ultimately confirmed or refuted.

The evolution of morphological novelties is frequently based on already existing gene regulatory networks (GRNs). As components of a single GRN are interconnected, differences in spatio-temporal expression of particular regulatory gene can potentially affect its downstream effectors, allowing for their redeployment in the novel context. The examples of such evolutionary co-options are widespread in both animals [63–65] and plants [66–68]. As FUMs might evolve through heterochronic changes in FM genetic patterning, the co-option of multiple components of flower-specific GRN can tentatively explain the origin of some novelties associated with floral units, including formation of pseudanthia. In all hitherto published studies, including our own (Fig. 10), expression patterns that lead to the establishment of ray flowers were recovered for genes that normally participate either in the specification of identity or symmetry of floral organs [18, 69–72]. Most of those transcription factors, especially CYC/TB1 genes are known to have independently undergone major expansion in several plant lineages with FUM-derived floral pseudanthia [73–77]. The expression of *CYC*-like genes is also documented in shoot-derived pseudocorollas of *Actinodium cunninghamii* [78]. Interestingly, petaloid bracts found in several plant lineages with floral units, such as *Nyssaceae* [79], *Cornaceae* [80], are patterned by the expression of B-class MADS box genes [81, 82] which normally specify the identity of true petals. Moreover, in dove tree (*Davidia involucrata*), the origin of petaloid bracts can be traced to duplication and neofunctionalization of *SUPPRESSOR OF OVEREXPRESSION OF CONSTANS1* [83]—a universal flowering pathway regulator [84]. Bract-derived pseudcorollas are also known to have evolved independently in numerous clades of Apioideae [29], and in some earlier-diverging umbellifer subfamilies,i.e. species of *Alepidea and Astrantia* from Saniculoideae, *Pozoa coriacea* from Azorelloideae and species of *Actintous* and *Xanthosia* from Mackinlayoideae [85, 86]. Although developmental data are scarce in Apiales, based on the occurrence of the umbel as the basic architectural model in reproductive shoots [87–89], we expect floral units to have evolved before the divergence of the clade uniting Apiaceae, Araliaceae and Myodocarpaceae [90, 91]. Later, the whole-genome duplication in the common ancestor of Apiaceae lead to the expansion of TCP gene family [92] which might have allowed for repeated co-option of newly acquired paralogues into the patterning of floral pseudanthia.

## Conclusions

The results presented in this study provide further evidence that the umbel in Apiaceae, the eponym of its traditional family name Umbelliferae or ‘umbel-bearing plants’, should be interpreted as a floral unit, i.e. a multiflowered iterative structure with flower-like characteristics. Its development illustrates all characteristics of floral unit meristems (FUM) including determinacy resulting from flower-like genetic patterning and morphogenesis driven by the processes of expansion and fractionation (self-organizing space-dependent process). As exemplified by surveys of various plant groups, these specific preconditions may constitute an important factor that drives the convergent evolution of pseudanthia. In future, we should aim to identify floral units across angiosperms to gain insight into the diversity of their patterning mechanisms. This in turn, may facilitate understanding of the evolution of plant reproductive structures and shed light on the origin of various morphological novelties associated with them.

## Materials and methods

### Plant material

Wild carrot (*Daucus carota* subsp. *carota*) was chosen as the model for investigating gene expression patterns in pseudanthia of Apiaceae. It is a biennial with relatively large and easy-to-manipulate umbels and one of the two umbellifers (the other being coriander) with available genomic data [93, 94]. All plants were sampled from natural populations in Warsaw, Poland (52.212671 N, 20.985909 E) and Mainz, Germany (49.992750 N, 8.244798 E). Dissected buds were preserved in cold FAA (50% ethanol, 5% (v/v) acetic acid and 3.7% (v/v) formaldehyde) and briefly vacuum-infiltrated several times. Subsequently, after dehydration and clearing performed according to the protocol of Karlgren et al. [95], the tissues were embedded in ParaPlast Plus^®^ blocks and stored at 4 ºC until further use. Additionally, a sample of dissected buds was chilled on ice and immediately used for RNA isolation. Morphological analysis was conducted on terminal and first-order umbels of six representatives of Apiaceae (Fig. 1) encompassing both phylogenetic and architectural diversity of pseudanthia in the family [29]. The list of vouchers is provided in Table 1.

**Table 1:** Species used in morphological investigation. Specimens and their respective vouchers are deposited at the herbaria: ADO and MJG

Species	Locality	Date	Collector(s)
*Artedia squamata* L	Turkey: Ankara, Campus of the Middle East Technical University	13.06.2012	F. Celep & R. Claßen-Bockhoff
*Coriandrum sativum* L	Germany: Mainz, cultivated in botanical garden at JGU	25.06.2001	R. Claßen-Bockhoff
*Echinophora trichophylla* Sm	Turkey: Bilecik, Küplü village, cemetery hill	19.04.2019	F. Celep & J. Baczynski
*Scandix pecten-veneris* L	Turkey: Anatalya, between villages Murtiçi and Gergles, roadside of Konya Manavgat Yolu	14.04.2019	F. Celep & J. Baczynski
*Tordylium apulum* L	Turkey: Anatalya, between villages Murtiçi and Gergles, roadside of Konya Manavgat Yolu	28.02.2019; 14.04.2019	F. Celep & J. Baczynski
*Tordylium brachytaenium* Boiss. & Heldr	Turkey: Antalya, rocky slopes of Tahtalı Mountains above Hurma village	15.04.2019	F. Celep & J. Baczynski

### Morphological investigation

After bud dissection (between 10 and 30 buds for each species), the specimens were immediately preserved in 70% ethanol. The probes were dehydrated in an ascending ethanol–acetone series, then were critically point-dried (BAL-TEC CPD030), sputter-coated with gold (BAL-TEC SCD005), and observed under the scanning electron microscope (ESEM XL-30 Philips). All steps were conducted according to the manufacturer’s protocols. The macrophotographs of carrot umbellets were taken using digital stereomicroscope Leica AM4815.

### Identification of genes of interest

The *CYC* orthologues are usually accompanied by numerous, highly similar paralogues. In order to isolate the genes of interest, we have retrieved all TCP family members from the genomes of: carrot (*D. carota,* NCBI: ASM162521v1), coriander (*Coriandrum sativum*, [94], available at http://cgdb.bio2db.com/), sunflower (*Helianthus annuus*, NCBI: GCA_002127325.2), black cottonwood (*Populus trichocarpa,* NCBI: GCA_000002775.3), blue columbine (*Aquilegia coerulea,* NCBI:GCA_002738505.1), beet (*Beta vulgaris,* NCBI: GCF_000511025.2)*,* wine (*Vitis vinifera,* NCBI: GCF_000003745.3)*,* thale cress (*Arabidopsis thaliana,* NCBI: TAIR10.1) and snapdragon (*Antirrhinum majus,* Li et al. 2019, available at http://bioinfo.sibs.ac.cn/Am/) with multiple BLASTP searches. The initial matrix totalling 360 gene accessions was aligned with MAFFT v.7.271 [96] using option *–auto* and manually trimmed to include only TCP and R domains. Sixty accessions belonging to *CYC*-like clade were then extracted from the aforementioned dataset, based on the presence of conserved threonine residues (Thr9 and Thr43, according to [97]). Phylogenetic inference for resulting matrix was conducted with maximum likelihood (ML) approach implemented in RAxML 8.2.4 with substitution model selected using PROTGAMMAAUTO algorithm [98]. Branch support was evaluated with 1000 rapid bootstrap replicates.

*LFY* and *UFO* orthologues (subsequently abbreviated as *DcLFY* and *DcUFO* for *D. carota*), are single-copy genes in vast majority of angiosperms [99–102]. Their retrieval was relatively straightforward with a use of protein BLAST [103]. To further confirm our identification, we conducted additional phylogenetic analyses with design similar to that described for TCP genes (Additional file 2).

### RNA extraction and in situ hybridization

Total RNA was extracted from dissected buds of *D. carota* with TRIzol^®^ reagent. The cDNA for probes synthesis was reverse transcribed using gene-specific primers with SuperScript IV First-Strand Synthesis System (ThermoFisher Scientific). Promoter for T7 or SP6 polymerase used for in vitro transcription of probes was introduced by PCR amplification with primers carrying appropriate overhangs in 5’ end (in forward primer for sense probe, in reverse for antisense, see Additional file 3). The gel-purified products were used in DIG-labelling reaction (DIG RNA labelling kit, Roche). Pretreatment, prehybridization, hybridization and posthybridization steps were performed according to Karlgren et al. [95] using 8–10 μm thick rotary microtome sections (Reichert OmS serial sections). Stringency washes were modified after Linke et al. [104]. Detection of hybridization reaction was performed with DIG nucleic acid detection kit (Roche), according to the manufacturer’s instructions.

## Supplementary Information

**Additional file 1**. Morphogenesis of high-order umbel in *Daucus carota*. **A **The incipient FUM is smaller than in terminal and first-order umbels and fractionates less umbellet meristems. **B **Umbellets initiate in a centripetal sequence – no clear division between peripheral and central umbellets can be seen. **C **The intermediate promotion of the umbel becomes apparent. Note three large peripheral umbellets with three ray flowers each and a smaller subperipheral umbellet with a single ray flower (in the bottom-right side of the photograph). **D **Ray flower meristems fractionate floral organs in a group-like pattern. **E **Mature peripheral umbellet with three distinct ray flowers subtended by pinnatisect involucels. **F **Subperipheral umbellet with a single, weak ray flower subtended by a asymmetrically bifid involucel. **G **Central umbellet with radial flowers and needle-like involucels. Abbreviations: irb – involucral bract, umbt – umbellet, ilb – involucellar bract; rafl – radial flower; ryfl – ray. Number next to abbreviation of floral organs denotes order of initiation. Scale bars = 100 µm.

**Additional file 2**. Maximum-likelihood tree of LFY and UFO genes based on their amino acid sequences. Major clades are defined with reference to canonical sequences. The sequences of DcLFY and DcUFO are marked with red. For visualization both trees were rooted with accessions of orthologues from Aquilegia coerulea (early-diverging eudicot). Bootstrap values < 70% were not plotted.

**Additional file 3**. Primers used for probes synthesis during mRNA *in situ *hybridization experiments.

## Data Availability

The datasets supporting the conclusions of this article are included within the article and its additional files or available from the TreeBase repository (study accession number: S29534).

## References

[1] Harder LD, Jordan CY, Gross WE, Routley MB (2004). Beyond floricentrism: the pollination function of inflorescences. Plant Species Biol.

[2] Ollerton J, Winfree R, Tarrant S (2011). How many flowering plants are pollinated by animals?. Oikos.

[3] Claßen-Bockhoff R (1990). Pattern analysis in pseudanthia. Plant Syst Evol.

[4] Classen-Bockhoff R (1992). Disposition, variation und Bewährung am Beispiel der Infloreszenzblumenbildung. Mitteilungen Aus Dem Hambg Zool Mus Inst.

[5] Endress PK (2010). Flower structure and trends of evolution in eudicots and their major subclades. Ann Mo Bot Gard.

[6] Jahnke C (1983). The development of the petals in the Apiaceae with special reference to the lobulum-inflexum. Acta Bot Neerlandica.

[7] Jahnke C, Froebe HA. Untersuchungen zur ontogenie des lobulum inflexum ausgewahlter Apiaceen-petalen. Beitrage Zur Biol Pflanz. 1984

[8] Froebe HA (1980). Randmusterbildung und synorganisation bei strahlenden Apiaceendolden. Plant Syst Evol.

[9] Ajani Y, Bull-Hereñu K, Claßen-Bockhoff R (2016). Patterns of flower development in Apiaceae-Apioideae. Flora.

[10] Prusinkiewicz P, Erasmus Y, Lane B, Harder LD, Coen E (2007). Evolution and development of inflorescence architectures. Science.

[11] Zhong J, Kong F (2021). The control of compound inflorescences: insights from grasses and legumes. Trends Plant Sci.

[12] Classen-Bockhoff R (2016). The shoot concept of the flower: still up to date?. Flora.

[13] Claßen-Bockhoff R, Bull-Hereñu K (2013). Towards an ontogenetic understanding of inflorescence diversity. Ann Bot.

[14] Runions A, Smith RS, Prusinkiewicz P (2014). Computational models of auxin-driven development. Auxin and its role in plant development.

[15] Zhang T, Cieslak M, Owens A, Wang F, Broholm SK, Teeri TH (2021). Phyllotactic patterning of gerbera flower heads. Proc Natl Acad Sci.

[16] Zhang T, Elomaa P (2021). Don’t be fooled: false flowers in Asteraceae. Curr Opin Plant Biol.

[17] Naghiloo S, Classen-Bockhoff R (2017). Understanding the unique flowering sequence in *Dipsacus fullonum*: evidence from geometrical changes during head development. PLoS ONE.

[18] Berger BA, Thompson V, Lim A, Ricigliano V, Howarth DG (2016). Elaboration of bilateral symmetry across Knautia macedonica capitula related to changes in ventral petal expression of CYCLOIDEA-like genes. EvoDevo.

[19] Carlson SE, Howarth DG, Donoghue MJ (2011). Diversification of *CYCLOIDEA*-like genes in Dipsacaceae (Dipsacales): implications for the evolution of capitulum inflorescences. BMC Evol Biol.

[20] Lu Z, Xu J, Li W, Zhang L, Cui J, He Q (2017). Transcriptomic analysis reveals mechanisms of sterile and fertile flower differentiation and development in *Viburnum macrocephalum f. keteleeri*. Front Plant Sci.

[21] Zhao Y, Zhang T, Broholm SK, Tähtiharju S, Mouhu K, Albert VA (2016). Evolutionary co-option of floral meristem identity genes for patterning of the flower-like Asteraceae inflorescence. Plant Physiol.

[22] Wilkinson MD, Haughn GW (1995). UNUSUAL FLORAL ORGANS controls meristem identity and organ primordia fate in Arabidopsis. Plant Cell.

[23] Fambrini M, Pugliesi C (2017). CYCLOIDEA 2 clade genes: key players in the control of floral symmetry, inflorescence architecture, and reproductive organ development. Plant Mol Biol Report.

[24] Broholm SK, Teeri TH, Elomaa P, Fornara F (2014). Molecular control of inflorescence development in *Asteraceae*. Advances in botanical research.

[25] Chapman MA, Leebens-Mack JH, Burke JM (2008). Positive selection and expression divergence following gene duplication in the sunflower *CYCLOIDEA* gene family. Mol Biol Evol.

[26] Chen J, Shen CZ, Guo YP, Rao GY (2018). Patterning the Asteraceae Capitulum: duplications and differential expression of the flower symmetry CYC2-like genes. Front Plant Sci.

[27] Gillies AC, Cubas P, Coen ES, Abbott RJ (2002). Making rays in the *Asteraceae*: genetics and evolution of radiate versus discoid flower heads. Dev Genet Plant Evol.

[28] Bull-Hereñu K, Claßen-Bockhoff R (2013). Testing the ontogenetic base for the transient model of inflorescence development. Ann Bot.

[29] Baczyński J, Sauquet H, Spalik K (2022). Exceptional evolutionary lability of flower-like inflorescences (pseudanthia) in *Apiaceae* subfamily *Apioideae*. Am J Bot.

[30] Pozner R, Zanotti C, Johnson LA (2012). Evolutionary origin of the Asteraceae capitulum: insights from Calyceraceae. Am J Bot.

[31] Philipson WR (1953). The relationships of the Compositae particularly as illustrated by the morphology of the inflorescence in the Rubiales and the Campanulatae. Phytomorphology.

[32] Small J (1918). The origin and development of the Compositae. New Phytol.

[33] Harris EM (1995). Inflorescence and floral ontogeny in *Asteraceae*: a synthesis of historical and current concepts. Bot Rev.

[34] Froebe HA. Inflorescence structure and evolution in Umbelliferae. In: The Biology and Chemistry of Umbelliferae. Cambridge: Academic Press; 1971. pp. 157–76.

[35] Weberling F (1992). Morphology of flowers and inflorescences.

[36] Kwiatkowska D (2008). Flowering and apical meristem growth dynamics. J Exp Bot.

[37] Claßen-Bockhoff R, Frankenhäuser H (2020). The ‘male flower’ of *Ricinus communis* (*Euphorbiaceae*) interpreted as a multi-flowered unit. Front Cell Dev Biol.

[38] Prusinkiewicz P, Barbier de Reuille P (2010). Constraints of space in plant development. J Exp Bot.

[39] Iwamoto A, Ishigooka S, Cao L, Ronse De Craene LP (2020). Floral development reveals the existence of a fifth staminode on the labellum of basal Globbeae. Front Ecol Evol.

[40] Bull-Hereñu K, Ronse De Craene LP (2020). Ontogenetic base for the shape variation of flowers in *Malesherbia* Ruiz & Pav. (*Passifloraceae*). Front Ecol Evol.

[41] El ES, Remizowa MV, Sokoloff DD (2020). Developmental flower and rhizome morphology in *Nuphar* (*Nymphaeales*): an interplay of chaos and stability. Front Cell Dev Biol.

[42] Nakagawa A, Kitazawa MS, Fujimoto K (2020). A design principle for floral organ number and arrangement in flowers with bilateral symmetry. Development.

[43] Fal K, Landrein B, Hamant O (2016). Interplay between miRNA regulation and mechanical stress for CUC gene expression at the shoot apical meristem. Plant Signal Behav.

[44] Landrein B, Kiss A, Sassi M, Chauvet A, Das P, Cortizo M (2015). Mechanical stress contributes to the expression of the STM homeobox gene in *Arabidopsis* shoot meristems. Elife.

[45] Palmer JH, Marc J (1982). Wound-induced initiation of involucral bracts and florets in the developing sunflower inflorescence. Plant Cell Physiol.

[46] Claßen-Bockhoff R (1992). Florale differenzierung in komplex organisierten Asteraceenköpfen. Flora.

[47] Owens A, Cieslak M, Hart J, Claßen-Bockhoff R, Prusinkiewicz P (2016). Modeling dense inflorescences. ACM Trans Graph.

[48] Claßen-Bockhoff R (1996). Functional units beyond the level of the capitulum and cypsela in Compositae. Compos Biol Util.

[49] Chandler JW (2014). Patterns and polarity in floral meristem and floral organ initiation. Crit Rev Plant Sci.

[50] Ronse De Craene L (2018). Understanding the role of floral development in the evolution of angiosperm flowers: clarifications from a historical and physico-dynamic perspective. J Plant Res.

[51] Naghiloo S, Dadpour MR, Movafeghi A (2012). Floral ontogeny in Astragalus compactus (Leguminosae: Papilionoideae: Galegeae): variable occurrence of bracteoles and variable patterns of sepal initiation. Planta.

[52] Tucker SC (1991). Helical floral organogenesis in *Gleditsia*, a primitive caesalpinioid legume. Am J Bot.

[53] Tucker SC (2000). Floral development and homeosis in *Saraca* (Leguminosae: Caesalpinioideae: Detarieae). Int J Plant Sci.

[54] Tucker SC (2001). The ontogenetic basis for missing petals in *Crudia* (Leguminosae: *Caesalpinioideae*: Detarieae). Int J Plant Sci.

[55] von Balthazar M, Endress PK (1999). Floral bract function, flowering process and breeding systems of *Sarcandra* and *Chloranthus* (*Chloranthaceae*). Plant Syst Evol.

[56] Bull-Hereñu K, Dos Santos P, Toni JFG, El Ottra JHL, Thaowetsuwan P, Jeiter J (2022). Mechanical forces in floral development. Plants.

[57] Naghiloo S (2020). Patterns of symmetry expression in angiosperms: developmental and evolutionary lability. Front Ecol Evol.

[58] Schoof H, Lenhard M, Haecker A, Mayer KF, Jürgens G, Laux T (2000). The stem cell population of Arabidopsis shoot meristems is maintained by a regulatory loop between the CLAVATA and WUSCHEL genes. Cell.

[59] Leibfried A, To JP, Busch W, Stehling S, Kehle A, Demar M (2005). WUSCHEL controls meristem function by direct regulation of cytokinin-inducible response regulators. Nature.

[60] Lenhard M, Bohnert A, Jürgens G, Laux T (2001). Termination of stem cell maintenance in Arabidopsis floral meristems by interactions between WUSCHEL and AGAMOUS. Cell.

[61] Levin JZ, Meyerowitz EM (1995). UFO: an Arabidopsis gene involved in both floral meristem and floral organ development. Plant Cell.

[62] Samach A, Klenz JE, Kohalmi SE, Risseeuw E, Haughn GW, Crosby WL (1999). The UNUSUAL FLORAL ORGANS gene of Arabidopsis thaliana is an F-box protein required for normal patterning and growth in the floral meristem. Plant J.

[63] Glassford WJ, Johnson WC, Dall NR, Smith SJ, Liu Y, Boll W (2015). Co-option of an ancestral hox-regulated network underlies a recently evolved morphological novelty. Dev Cell.

[64] Hu Y, Schmitt-Engel C, Schwirz J, Stroehlein N, Richter T, Majumdar U (1885). A morphological novelty evolved by co-option of a reduced gene regulatory network and gene recruitment in a beetle. Proc R Soc B.

[65] Yamazaki A, Yamakawa S, Morino Y, Sasakura Y, Wada H (2021). Gene regulation of adult skeletogenesis in starfish and modifications during gene network co-option. Sci Rep.

[66] Baxter CEL, Costa MMR, Coen ES (2007). Diversification and co-option of *RAD*-like genes in the evolution of floral asymmetry. Plant J.

[67] de Almeida AMR, Yockteng R, Schnable J, Alvarez-Buylla ER, Freeling M, Specht CD (2014). Co-option of the polarity gene network shapes filament morphology in angiosperms. Sci Rep.

[68] Gupta MD, Tsiantis M (2018). Gene networks and the evolution of plant morphology. Curr Opin Plant Biol.

[69] Garcês HMP, Spencer VM, Kim M (2016). Control of floret symmetry by *RAY3*, *SvDIV1B*, and *SvRAD* in the capitulum of *Senecio vulgaris*. Plant Physiol.

[70] Shen CZ, Chen J, Zhang CJ, Rao GY, Guo YP (2021). Dysfunction of *CYC2g* is responsible for evolutionary shift from radiate to disciform flowerhead in the *Chrysanthemum* group (*Asteraceae*: *Anthemideae*). Plant J.

[71] Zhang T, Zhao Y, Juntheikki I, Mouhu K, Broholm SK, Rijpkema AS (2017). Dissecting functions of *SEPALLATA*-like MADS box genes in patterning of the pseudanthial inflorescence of *Gerbera hybrida*. New Phytol.

[72] Zhao Y, Broholm SK, Wang F, Rijpkema AS, Lan T, Albert VA (2020). TCP and MADS-box transcription factor networks regulate heteromorphic flower type identity in *Gerbera hybrida*. Plant Physiol.

[73] Boyden GS, Donoghue MJ, Howarth DG (2012). Duplications and expression of *RADIALIS*-like genes in Dipsacales. Int J Plant Sci.

[74] Hegarty MJ, Jones JM, Wilson ID, Barker GL, Coghill JA, Sanchez-Baracaldo P (2005). Development of anonymous cDNA microarrays to study changes to the Senecio floral transcriptome during hybrid speciation. Mol Ecol.

[75] Ruokolainen S, Ng YP, Albert VA, Elomaa P, Teeri TH (2010). Large scale interaction analysis predicts that the *Gerbera hybrida* floral E function is provided both by general and specialized proteins. BMC Plant Biol.

[76] Tähtiharju S, Rijpkema AS, Vetterli A, Albert VA, Teeri TH, Elomaa P (2012). Evolution and diversification of the CYC/TB1 gene family in *Asteraceae*—a comparative study in *Gerbera* (Mutisieae) and sunflower (Heliantheae). Mol Biol Evol.

[77] Zahn LM, Kong H, Leebens-Mack JH, Kim S, Soltis PS, Landherr LL (2005). The evolution of the *SEPALLATA* subfamily of MADS-box genes: a preangiosperm origin with multiple duplications throughout angiosperm history. Genetics.

[78] Claßen-Bockhoff R, Ruonala R, Bull-Hereñu K, Marchant N, Albert VA (2013). The unique pseudanthium of *Actinodium* (Myrtaceae)-morphological reinvestigation and possible regulation by CYCLOIDEA-like genes. EvoDevo.

[79] Claßen-Bockhoff R, Arndt M (2018). Flower-like heads from flower-like meristems: pseudanthium development in *Davidia involucrata* (Nyssaceae). J Plant Res.

[80] Liu J, Franks RG, Feng CM, Liu X, Fu CX, Xiang QY (2013). Characterization of the sequence and expression pattern of LFY homologues from dogwood species (Cornus) with divergent inflorescence architectures. Ann Bot.

[81] Feng CM, Liu X, Yu Y, Xie D, Franks RG, Xiang QYJ (2012). Evolution of bract development and B-class MADS box gene expression in petaloid bracts of Cornus s. l. (*Cornaceae*). New Phytol.

[82] Vekemans D, Viaene T, Caris P, Geuten K (2012). Transference of function shapes organ identity in the dove tree inflorescence. New Phytol.

[83] Li G, Cao C, Yang H, Wang J, Wei W, Zhu D (2020). Molecular cloning and potential role of DiSOC1s in flowering regulation in *Davidia involucrata* Baill. Plant Physiol Biochem.

[84] Moon J, Suh SS, Lee H, Choi KR, Hong CB, Paek NC (2003). The SOC1 MADS-box gene integrates vernalization and gibberellin signals for flowering in Arabidopsis. Plant J.

[85] Froebe HA. Die Blütenstände der Saniculoideen (Umbelliderae): eine vergleichend-morphologische und entwicklungsgeschichtliche Untersuchung. 1964.

[86] Froebe HA (1979). Die Infloreszenzen der Hydrocotyloideen (Apiaceae). Trop Subtrop Pflanzenwelt.

[87] Plunkett GM, Wen J, Lowry PP, Mitchell AD, Henwood MJ, Fiaschi P, Kadereit JW, Bittrich V (2018). Araliaceae. Flowering plants eudicots.

[88] Lowry PP, Plunkett GM, Kadereit JW, Bittrich V (2018). Myodocarpaceae. Flowering plants eudicots.

[89] Plunkett GM, Pimenov MG, Reduron JP, Kljuykov EV, Lee BY, van Wyk BE, Kubitzky K (2018). Apiaceae. The families and genera of vascular plants.

[90] Chandler GT, Plunkett GM (2004). Evolution in Apiales: nuclear and chloroplast markers together in (almost) perfect harmony. Bot J Linn Soc.

[91] Nicolas AN, Plunkett GM (2014). Diversification times and biogeographic patterns in Apiales. Bot Rev.

[92] Pei Q, Li N, Bai Y, Wu T, Yang Q, Yu T (2021). Comparative analysis of the TCP gene family in celery, coriander and carrot (family *Apiaceae*). Veg Res.

[93] Iorizzo M, Ellison S, Senalik D, Zeng P, Satapoomin P, Huang J (2016). A high-quality carrot genome assembly provides new insights into carotenoid accumulation and asterid genome evolution. Nat Genet.

[94] Song X, Nie F, Chen W, Ma X, Gong K, Yang Q (2020). Coriander genomics database: a genomic, transcriptomic, and metabolic database for coriander. Hortic Res.

[95] Karlgren A, Carlsson J, Gyllenstrand N, Lagercrantz U, Sundström JF (2009). Non-radioactive in situ hybridization protocol applicable for Norway spruce and a range of plant species. J Vis Exp JoVE.

[96] Katoh K, Standley DM (2013). MAFFT multiple sequence alignment software version 7: improvements in performance and usability. Mol Biol Evol.

[97] González-Grandío E, Cubas P. TCP Transcription factors: evolution, structure, and biochemical function. In: plant transcription factors. Elsevier; p. 139–51. 2016. https://www.linkinghub.elsevier.com/retrieve/pii/B9780128008546000099. Accessed 10 Feb 2022

[98] Stamatakis A (2014). RAxML version 8: a tool for phylogenetic analysis and post-analysis of large phylogenies. Bioinformatics.

[99] Moyroud E, Kusters E, Monniaux M, Koes R, Parcy F (2010). LEAFY blossoms. Trends Plant Sci.

[100] Moyroud E, Tichtinsky G, Parcy F (2009). The LEAFY floral regulators in angiosperms: conserved proteins with diverse roles. J Plant Biol.

[101] Zhang X, González-Carranza Z, Zhang S, Miao Y, Liu CJ, Roberts J. F-Box Proteins in Plants. In 2019. p. 1–21.

[102] Abd-Hamid NA, Ahmad-Fauzi MI, Zainal Z, Ismail I (2020). Diverse and dynamic roles of F-box proteins in plant biology. Planta.

[103] Johnson M, Zaretskaya I, Raytselis Y, Merezhuk Y, McGinnis S, Madden TL (2008). NCBI BLAST: a better web interface. Nucleic Acids Res.

[104] Linke B, Nothnagel T, Börner T (2003). Flower development in carrot CMS plants: mitochondria affect the expression of MADS box genes homologous to *GLOBOSA* and *DEFICIENS*. Plant J.

