# Supplementary material for: Flower-like meristem conditions and spatial constraints shape architecture of floral pseudanthia in Apioideae
Source: EvoDevo. 2022 Dec 19;13:19. doi: 10.1186/s13227-022-00204-6 (PMC9764545; doi:10.1186/s13227-022-00204-6)
Supplement: Supplementary file 1 — Additional file 1. Morphogenesis of high-order umbel in Daucus carota. A The incipient FUM is smaller than in terminal and first-order umbels and fractionates less umbellet meristems. B Umbellets initiate in a centripetal sequence – no clear division between peripheral and central umbellets can be seen. C The intermediate promotion of the umbel becomes apparent. Note three large peripheral umbellets with three ray flowers each and a smaller subperipheral umbellet with a single ray flower (in the bottom-right side of the photograph). D Ray flower meristems fractionate floral organs in a group-like pattern. E Mature peripheral umbellet with three distinct ray flowers subtended by pinnatisect involucels. F Subperipheral umbellet with a single, weak ray flower subtended by a asymmetrically bifid involucel. G Central umbellet with radial flowers and needle-like involucels. Abbreviations: irb – involucral bract, umbt – umbellet, ilb – involucellar bract; rafl – radial flower; ryfl – ray. Number next to abbreviation of floral organs denotes order of initiation. Scale bars = 100 µm. [file 13227_2022_204_MOESM1_ESM.docx]

**Additional file 1**


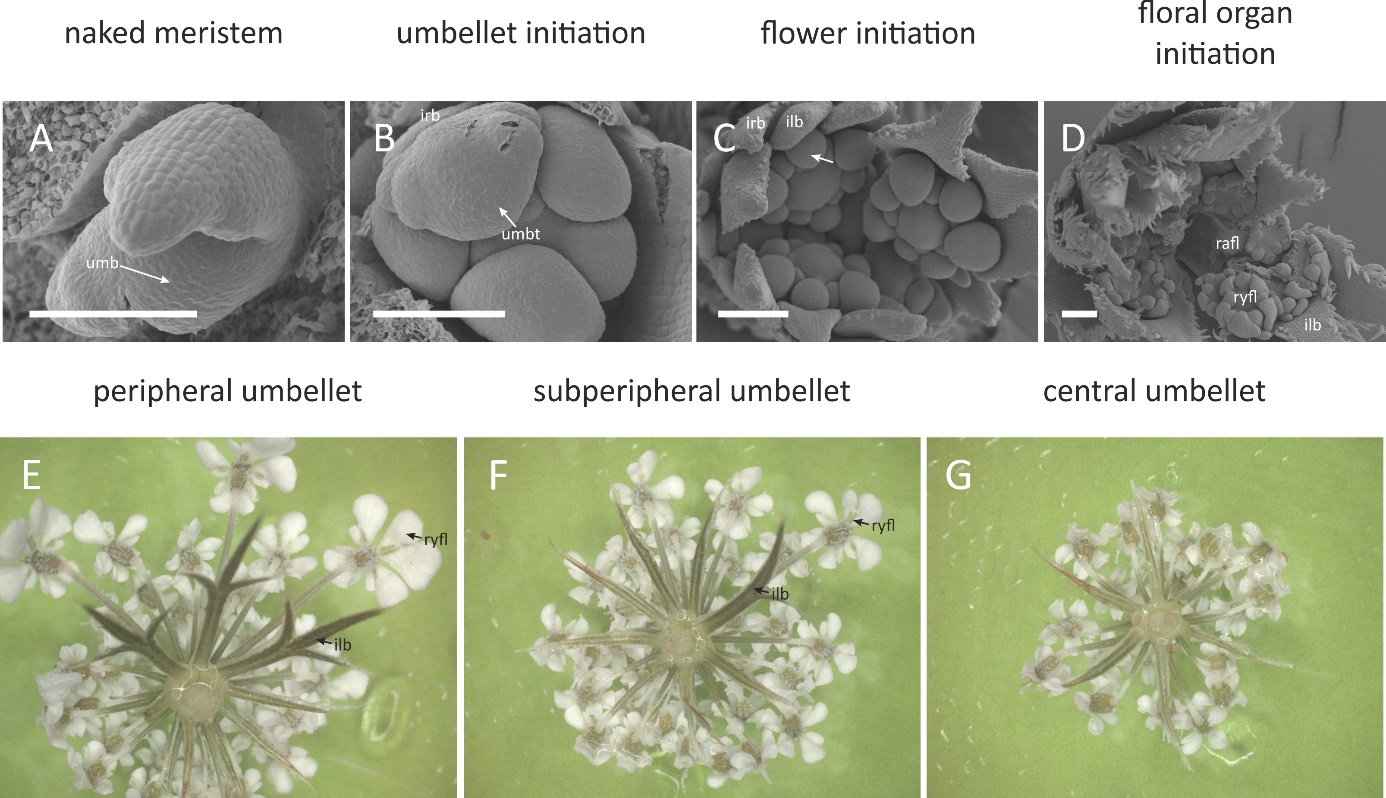


Morphogenesis of high-order umbel in *Daucus carota*. **A** The incipient FUM is smaller than in terminal and first-order umbels and fractionates less umbellet meristems **B** Umbellets initiate in a centripetal sequence – no clear division between peripheral and central umbellets can be seen. **C** The intermediate promotion of umbel becomes apparent. Note three large peripheral umbellets with three ray flowers each and a smaller subperipheral umbellet with a single ray flower (in the bottom-right side of the photograph) **D** Ray flower meristems fractionate floral organs in a group-like pattern. **E** Mature peripheral umbellet with three distinct ray flowers subtended by pinnatisect involucels **F** Subperipheral umbellet with a single, weak ray flower subtended by a asymmetrically bifid involucel. **G** Central umbellet with radial flowers and needle-like involucels. Abbreviations: irb – involucral bract, umbt – umbellet, ilb – involucellar bract; rafl – radial flower; ryfl – ray. Number next to abbreviation of floral organs denotes order of initiation. Scale bars = 100 µm.
