# Supplementary material for: Flower-like meristem conditions and spatial constraints shape architecture of floral pseudanthia in Apioideae
Source: EvoDevo. 2022 Dec 19;13:19. doi: 10.1186/s13227-022-00204-6 (PMC9764545; doi:10.1186/s13227-022-00204-6)
Supplement: Supplementary file 2 — Additional file 2. Maximum-likelihood tree of LFY and UFO genes based on their amino acid sequences. Major clades are defined with reference to canonical sequences. The sequences of DcLFY and DcUFO are marked with red. For visualization both trees were rooted with accessions of orthologues from Aquilegia coerulea (early-diverging eudicot). Bootstrap values < 70% were not plotted. [file 13227_2022_204_MOESM2_ESM.docx]

**Additional file 3**


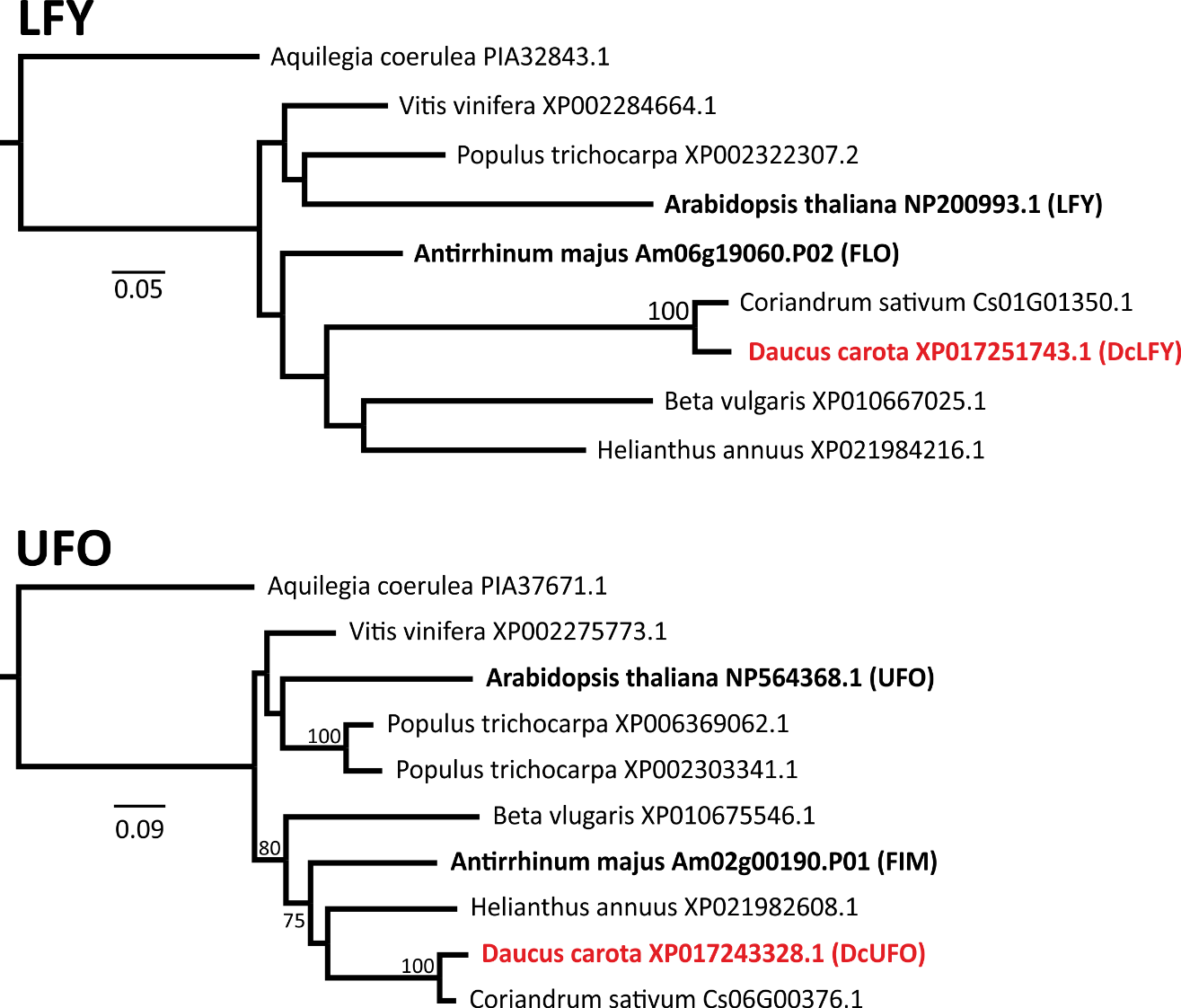


Maximum-likelihood tree of LFY (upper) and UFO (lower) genes based on their amino-acid sequences. Major clades are defined with reference to canonical sequences. The sequences of *DcLFY* and *DcUFO* are marked with red. Both trees are rooted with accessions orthologues from *Aquilegia coerulea* (early-diverging eudicot). Bootstrap values < 70% were not plotted.
